# Increased Expression of Desmin and Vimentin Impairs Mitochondrial Function and Reduces Contractility via G0S2 in Bladder Smooth Muscle

**DOI:** 10.1096/fj.202502912R

**Published:** 2026-10-21

**Authors:** Elham Javed, Chellappagounder Thangavel, Karthik Thangavel, Joseph Hypolite, Isabelle Gamache, Yilin Wang, Sankar Addya, Darin Mak, Furqan Syed, Jyotsna Mishra, Vilas Desai, Pragnya Das, Dominic R. Villalba, Alok Bhushan, Shey‐Shing Sheu, Sunday A. Shoyele, Erin L. Seifert, Jose G. Teodoro, Steven S. An, Raymond B. Penn, Deepak A. Deshpande, Ettickan Boopathi

**Affiliations:** ^1^ Department of Medicine Center for Translational Medicine Philadelphia Pennsylvania USA; ^2^ Department of Pharmaceutical Science, Jefferson College of Pharmacy Thomas Jefferson University Philadelphia Pennsylvania USA; ^3^ Department of Biochemistry, Rosalind and Morris Goodman Cancer Institute McGill University Montréal Quebec Canada; ^4^ Department of Biochemistry McGill University Montréal Quebec Canada; ^5^ Cancer Genomics Thomas Jefferson University Philadelphia Pennsylvania USA; ^6^ Department of Pharmacology, Rutgers Institute for Translational Medicine and Science Rutgers‐Robert Wood Johnson Medical School New Brunswick New Jersey USA; ^7^ Cooper University Hospital Camden New Jersey USA; ^8^ Department of Pathology and Genomic Medicine Thomas Jefferson University Philadelphia Pennsylvania USA

**Keywords:** bladder smooth muscle, contraction, desmin, G0S2, mitochondria, phospho JNK, phospho MLC20, vimentin

## Abstract

Bladder outlet obstruction caused by benign prostatic hyperplasia in humans, as well as partial bladder outlet obstruction in animal models, is linked to reduced contractility and impaired mitochondrial function in bladder smooth muscle (BSM). However, the mechanisms underlying changes in BSM contractility and mitochondrial function are still largely unknown. Our previous study showed that increased expression of intermediate filament proteins desmin and vimentin weakens BSM contractility. In this study, we hypothesize that BSM contractile dysfunction, driven by elevated levels of desmin and vimentin, is associated with impaired mitochondrial function. Human BSM cells and murine BSM tissue with elevated levels of desmin and vimentin produce less force than control BSM. Notably, increased expression of desmin and vimentin in human BSM cells reduces mitochondrial elongation and branching, with associated decreases in the fusion proteins MFN1 and MFN2, oxygen consumption rate, and ATP levels, and an increase in mitochondrial reactive oxygen species. Microarray analysis revealed significant induction of G0/G1 switch gene 2 (G0S2) mRNA expression in desmin‐ and vimentin‐overexpressing human BSM cells. G0S2 protein interacts with mitochondrial channel protein VDAC in desmin‐ and vimentin‐overexpressing human BSM cells. Silencing G0S2 expression restored BSM contraction, MLC20 phosphorylation, and ATP levels, whereas reducing the mitochondrial ROS and phospho‐JNK levels in human BSM cells and murine BSM tissue overexpressing desmin and vimentin. Overall, these findings show that G0S2 plays a critical role in desmin‐ or vimentin‐mediated attenuation of BSM contraction and mitochondrial dysfunction.

## Graphical abstract

Our previous study demonstrated that elevated levels of desmin and vimentin reduce BSM contractility. This study shows that the reduced BSM contractility is associated with mitochondrial impairment. G0/G1 switch gene 2 (G0S2) is upregulated in desmin‐ and vimentin‐overexpressing BSM. Silencing G0S2 expression restored BSM contractility, pMLC20, and mitochondrial function and reduced pJNK levels in cells overexpressing desmin and vimentin. These findings show that BSM contractile and mitochondrial dysfunction, driven by elevated levels of desmin and vimentin, are mediated by G0S2.

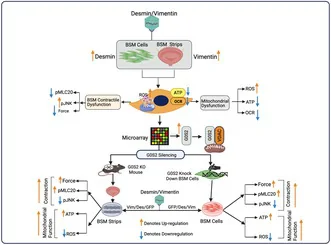

**Abbreviations**
AR: aspect ratio
BPH: benign prostatic hyperplasia
BSM: bladder smooth muscle
FF: form factor
G0S2: G0/G1 switch gene 2
IF: intermediate filament
JNK: c‐Jun N‐terminal kinase
MLC: myosin light chain
OCR: oxygen consumption rate
VDAC: voltage‐dependent anion channel

## 1 Introduction

Benign prostatic hyperplasia (BPH) is a common condition in aging men and a significant cause of lower urinary tract symptoms [1, 2, 3, 4]. It is also the leading cause of bladder outlet obstruction (BOO) [2, 3], which induces bladder dysfunction, urinary retention, and morphological changes, including smooth muscle hypertrophy and bladder wall fibrosis [5, 6, 7, 8]. Initially, bladder smooth muscle (BSM) cells undergo hypertrophy in response to pathological stimuli. However, prolonged stimulation results in contractile dysfunction [6, 9, 10, 11]. Studies using animal models of partial BOO and BPH‐induced BOO have demonstrated that BSM remodeling involves changes in the expression of contractile and regulatory proteins, as well as intermediate filament (IF) proteins desmin and vimentin [5, 7, 12, 13, 14, 15]. A previous study from our group demonstrated that the overexpression of IF proteins desmin and vimentin reduces BSM contractility via JNK2 in mice [16]. This strongly implicates an IF/JNK2‐dependent mechanism in BSM contractile dysfunction in BPH‐induced BOO.

Mitochondria are essential for energy metabolism, generating the majority of cellular ATP through oxidative phosphorylation and serving as the primary source of intracellular reactive oxygen species (ROS). They are involved in cell signaling and the maintenance of redox balance [17, 18]. BOO leads to impaired BSM mitochondrial function in both animal models and in patients with BPH [19, 20, 21, 22, 23]. The loss of mitochondrial morphology in BSM tissue is correlated with the urodynamic severity of BOO in patients with BPH [19, 24]. However, these studies do not address whether the specific induction of desmin and vimentin expression is sufficient to alter mitochondrial morphology or function, or the mechanisms by which IFs impact mitochondrial function. This question is particularly relevant, as previous studies have shown that mitochondria depend on IF proteins to maintain their structure and regulate cellular respiration [25].

Here, we hypothesize that BSM contractile dysfunction, driven by elevated levels of desmin and vimentin, is associated with impaired mitochondrial function. Our findings in the present study demonstrate that BSM with elevated expression of desmin and vimentin generates less force and exhibits reduced levels of phosphorylated MLC20 compared to control BSM. Increased expression of desmin and vimentin in BSM reduces mitochondrial elongation and branching, associated with reduced ATP levels and increased mitochondrial ROS accumulation. Unbiased microarray analysis revealed a significant increase in G0S2 mRNA expression. G0S2 protein interacts with voltage‐dependent anion channel (VDAC) in desmin‐ and vimentin‐overexpressing human BSM cells. Notably, silencing G0S2 expression in BSM restored contractile function, pMLC20 levels, and ATP levels, whereas reducing the elevated mitochondrial ROS and phospho‐JNK levels induced by desmin and vimentin overexpression. Collectively, these studies suggest that the mitochondrial protein G0S2 plays a critical role in desmin‐ or vimentin‐mediated attenuation of BSM contraction and mitochondrial dysfunction.

## 2 Materials and Methods

### 2.1 Chemicals and Buffers

Tyrode buffer (120 mM NaCl, 2.7 mM KCl, 23.8 mM NaHCO_3_, 0.5 mM MgCl_2_, 0.4 mM NaH_2_PO_4_, 1.8 mM CaCl_2_, and 5.6 mM dextrose; pH 7.4), physiological salt solution (140 mM NaCl, 4.7 mM KCl, 1.2 mM MgSO_4_, 1.6 mM CaCl_2_, 1.2 mM Na_2_HPO_4_, 2 mM MOPS (pH 7.4), 5 mM D‐glucose, and 0.02 mM EDTA), and RIPA lysis buffer (150 mM NaCl, 1.0% NP40, 0.5% deoxycholate, 0.1% SDS, 50 mM Tris (pH 8.0)) containing protease inhibitors and phosphatase inhibitors (Millipore Sigma, Missouri) were used in this study. The synthetic Arg‐Gly‐Asp (RGD)‐containing peptide was purchased from Biosynth (Gardner, MA).

### 2.2 Cell Culture, Transfection, and Transduction

Primary human BSM cells (Cat # No. CC‐2533) and smooth muscle cell growth medium‐2 (SmGM‐2) were obtained from Lonza (Walkersville, MD). These primary human BSM cells were isolated from human bladder tissue. SmGM‐2 (Cat # CC‐3182) contains basal medium supplemented with 5% FBS, 0.1% insulin, 0.1% hEGF, 0.2% hFGF‐B, and 0.1% Gentamicin Sulfate‐Amphotericin (GA‐1000). Human BSM cells were cultured in SmGM‐2 medium and transfected using the Amaxa Nucleofector II (Amaxa Inc., Gaithersburg, MD) and Basic SMC Nucleofector II kit (Lonza, Walkersville, MD) according to the manufacturer's protocol. Transfected cells were plated on 35‐mm MatTek dishes with a 1.5‐mm glass‐bottom coverslip (MatTek Corp., Ashland, MA). Primary human BSM cells were transduced with an adenovirus encoding GFP or desmin‐GFP, or vimentin‐GFP for 36 h. Cells were grown at 37°C in a humidified incubator containing 5% CO_2_. G0S2 gene silencing was achieved through lentiviral transduction of G0S2‐specific short hairpin RNA (shRNA) or a scrambled shRNA sequence (served as a control) in human BSM cells. For all experiments in this study, human BSM cells at passages 3–5 were used.

### 2.3 MnSOD Overexpression in Human BSM Cells

Superoxide dismutase 2 (MnSOD/SOD2) cDNA (Cat # HG12061‐CF; Sino Biological, Wayne, PA) or empty vector was transfected into human BSM cells using the Amaxa Basic SMC Nucleofector II kit according to the manufacturer's protocol. Overexpression of superoxide dismutase was confirmed by immunoblot analysis. Additionally, MnSOD activity was measured to confirm its enzymatic activity. To induce mitochondrial ROS, cells were treated with antimycin A (Sigma‐Aldrich) for 36 h. We treated the cells with increasing concentrations of antimycin A for 36 h to establish the highest concentration that preserved maximal cell viability. Concentrations up to 100 nM had a minimal effect on viability (more than 95% cell survival). Based on this data, we selected 100 nM of antimycin A as the maximum concentration for the subsequent experiment. Cell viability was assessed using a Trypan blue exclusion assay. Control cells received an equivalent volume of DMSO as a vehicle at a final concentration of ≤ 0.1%.

### 2.4 Immunoblot Analysis

Total proteins from human BSM cells were extracted using RIPA lysis buffer supplemented with phosphatase and protease inhibitors. Protein lysates were electrophoresed on a sodium dodecyl sulfate‐polyacrylamide (SDS‐PAGE) gel. Proteins were transferred onto a 0.45 μm polyvinylidene difluoride membrane (EMD Millipore, Burlington, MA) after electrophoresis, blocked with Tris‐buffered saline containing 5% dry milk and 0.05% Tween‐20, and probed with primary antibodies. After washing, the membranes were incubated with secondary HRP‐conjugated mouse, rabbit, and goat antibodies. The details of the antibody source are presented in Table S1. Immunoreactive protein signals were visualized with enhanced chemiluminescence, and band intensities were quantified using FIJI (ImageJ, NIH).

### 2.5 Measurement of MLC20 Phosphorylation

MLC20 phosphorylation in murine BSM strips was measured as previously described [16]. Briefly, BSM strips were stimulated with 120 mM KCl, then rapidly frozen in liquid nitrogen and immersed in a dry ice/acetone slurry containing 10% trichloroacetic acid (TCA) and 10 mM DTT. The strips were slowly thawed at room temperature, washed in acetone, air‐dried, homogenized, and the protein was precipitated with 10% TCA in the presence of 10 mM DTT. The samples were centrifuged at 12 000 rpm for 10 min, then resuspended in the sample buffer. Proteins were separated by SDS‐PAGE and then analyzed by immunoblotting. For the measurement of MLC20 phosphorylation in human BSM cells, cells were stimulated with 120 mM KCl for 10 min, followed by the addition of stop buffer (a 1:1 mixture of water and perchloric acid) for 10 min on ice. After centrifugation, the pellet was washed thoroughly with ice‐cold Tyrode buffer to remove excess perchloric acid. Subsequently, the cells were lysed in RIPA buffer containing protease and phosphatase inhibitors. Proteins were separated by SDS‐PAGE and then analyzed by immunoblotting.

### 2.6 Immunofluorescence and Confocal Microscopy

Human BSM cells were grown on collagen‐coated coverslips. Cells were infected with an adenovirus encoding GFP, desmin, or vimentin, and after 36 h post‐infection, they were fixed with 4% formaldehyde. The cells were permeabilized with 0.1% Triton‐X100, washed three times with PBS, and blocked with 0.25% BSA for 1 h at room temperature. Alexa Fluor 555 desmin antibody or Cy3‐labeled vimentin antibody was incubated overnight at 4°C. The coverslips were washed with TBST (Tris‐buffered saline containing Tween‐20) and mounted with VectaShield mounting medium (Vector Laboratories Inc., Burlingame, CA). Nuclei were stained with DAPI. Images were acquired using a Leica Stellaris 8 confocal microscope with Leica HyD‐S detectors. Confocal images were acquired using identical microscope settings, with autoscaling applied across groups. Auto‐scaled images were used for visualization and to determine the subcellular distribution. The image files were processed using a Python‐based pipeline to visualize the desmin and vimentin filament network.

### 2.7 Python‐Based Analysis of Desmin and Vimentin Filaments

Raw Greyscale confocal images were normalized to a 0–1 intensity range across the field of view. To reduce image noise, a Gaussian filter (*σ* = 1.0) was applied. Filamentous structures were segmented using Otsu's thresholding method to generate binary masks for subsequent analysis. Small objects and holes smaller than 50 pixels were removed using morphological filtering. Binary filament masks were skeletonized to generate reduced centerline representations of the filament network while preserving network connectivity.

### 2.8 Mitochondrial Morphology

Mitochondrial morphology was evaluated in human BSM cells expressing the mitochondria‐targeted red fluorescent protein, DsRed2‐Mito‐7. Human BSM cells were transfected via electroporation with DsRed2‐Mito‐7 (Addgene, MA) following the Amaxa Basic SMC Nucleofector II protocol (Lonza, USA). After electroporation, human BSM cells were plated on a glass‐bottom plate. After 24 h, the cells were transduced with adenovirus encoding GFP, desmin, and vimentin, followed by a 36‐h incubation. After a 36‐h incubation, cells were washed and imaged using a confocal microscope (FV500; Olympus, USA). DsRed2‐Mito‐7 was excited at 558 nm, and its emission was collected at 583 nm using a 60× oil‐immersion objective on the Nikon confocal microscope.

### 2.9 Python‐Based Analysis of Mitochondrial Morphology

#### 2.9.1 Dataset Preparation

We used a custom, *Python‐based pipeline* to extract morphological features of mitochondria, such as aspect ratio and form factor, from confocal fluorescent images [26]. Images were analyzed across the field of view, and all images were manually curated and then processed using a Python‐based script to generate CSV files for later analysis [27]. Image processing was carried out using open‐source Python libraries, including scikit‐image [28], OpenCV [29], NumPy, and Skan.

#### 2.9.2 Image Processing

Microscopic images were read using *skimage.io.imread* and converted to grayscale using *cv2.cvtColor*. To segment mitochondrial structures, we applied Otsu's adaptive thresholding method. The resulting binary mask served as the basis for both shape and skeleton analyzes. Binary images were labeled using *skimge.measure.label* to identify individual mitochondrial structures [28]. Morphological descriptions were extracted for each mitochondrial component using the regionprops function. We computed the aspect ratio (AR) and form factor (FF) to assess mitochondrial shape. AR is defined as the ratio of the lengths of the major and minor axes, with a value of 1 indicating a perfect circle and higher values indicating increased elongation (e.g., an AR of 3 indicates a mitochondrion three times longer than it is wide). FF is calculated as 4*π*·Area/Perimeter^2^ and serves as a measure of circularity.

#### 2.9.3 Skeleton‐Based Branching Analysis

We evaluated overall connectivity and the mitochondrial network using the skeletonized network. To capture mitochondrial branching, we skeletonized the binary masks using the skeletonize function from the *skimage.morphology* module. The skeletonized images were analyzed using the S*kan* library [30]. This library provides a graph‐based representation of the skeleton for computing path‐based metrics. The Skan analysis output provided quantitative information on mitochondrial connectivity and complexity [30], including the number of branches (total skeleton segments), the mean branch length (average length of skeleton paths), and the total branch length (cumulative length of all skeleton paths).

### 2.10 Transmission Electron Microscopy

Mitochondrial morphology and ultrastructural analysis of actomyosin filaments were performed using transmission electron microscopy (TEM). Human BSM cells were fixed in 2.5% glutaraldehyde in 0.1 M sodium cacodylate buffer (pH 7.4) at 4°C overnight, followed by washing in 0.1 M sodium cacodylate buffer (pH 7.4). Cells were then post‐fixed in 2% osmium tetroxide (OsO_4_) in 0.1 M sodium cacodylate buffer (pH 7.4) at 4°C for 1 h. Cells were washed three times in double‐distilled water and then incubated in 1% aqueous uranyl acetate for 15 min to enhance membrane contrast. Cells in agarose blocks were dehydrated using a graded series of acetone. Following dehydration, cells were infiltrated with graded acetone‐Spurr resin mixtures and embedded in fresh Spurr resin. Ultrathin sections were cut from resin‐embedded blocks and picked up on a copper grid. TEM was performed at the MitoCare Center Electron Microscopy Facility at Thomas Jefferson University using an FEI Tecnai 12 transmission electron microscope operated at 120 keV. The instrument has been upgraded with a tomography package using FEI Xplore 3D Tomography software. Mitochondria were imaged using an AMT XR111 CCD camera. Actin and myosin filaments were imaged using an AMT Bio Sprint 12 CCD camera. Actin and myosin filaments were identified by their characteristic sizes.

### 2.11 Measurement of Mitochondrial Reactive Oxygen Species in Intact Cells

Mitochondrial ROS levels were measured in live cells using the MitoSOX Red fluorescence dye. MitoSOX Red is a derivative of dihydroethidium that quickly accumulates in actively respiring mitochondria because it is covalently linked to the lipophilic cation triphenyl phosphonium ion, which helps MitoSOX enter mitochondria [31]. The oxidation of MitoSOX by superoxide results in the generation of 2‐hydroxyethidium, which is retained by mitochondria and displays red fluorescence [32]. To assess changes in mitochondrial ROS levels, human BSM cells were seeded in SmGM2 medium on a glass‐bottomed dish (MatTek Corp., Ashland, MA). Twenty‐four hours later, the cells were transduced with an adenovirus encoding GFP, desmin, and vimentin, and then incubated for an additional 36 h. After 36 h of incubation, cells were incubated with 5 μM of MitoSOX Red (Life Technologies, Carlsbad, CA) and 10 nM of MitoTracker Deep Red (Life Technologies) for 10 min at 37°C and 5% CO_2_. To verify the mitochondrial localization of MitoSOX, we employed a membrane‐potential‐insensitive mitochondrial‐targeted dye, MitoTracker Deep Red. MitoSOX and MitoTracker Deep Red were excited at 510 and 644 nm, respectively, and their emissions were collected at 580 and 665 nm, respectively. Live‐cell imaging was conducted using a Nikon A1R confocal microscope.

### 2.12 Mitochondrial ROS Quantitation Using a Python‐Based Pipeline

Fluorescence intensity of ROS in BSM cells was quantified across the field of view using a custom Python‐based image analysis pipeline built with NumPy, OpenCV, and scikit‐image. Microscopic images were loaded, and the fluorescence corresponding to the ROS signal was extracted for analysis. Background subtraction was then performed by estimating the background intensity using the 5th‐percentile pixel value for each image. This background value was subtracted from the image.

All negative values were clipped to zero. To identify ROS‐positive regions, images were normalized to the 0–1 intensity range and Thresholded at 0.1 to generate a binary mask. Small objects with fewer than 20 pixels were removed during design protocol filtering to reduce noise and exclude non‐specific signals. ROS quantification metrics were subsequently extracted from the threshold regions. ROS intensity was calculated as the sum of the background‐corrected pixel intensities within the binary mask. All images within each experimental condition were processed automatically, and the resulting measurements were compiled into CSV and Excel output files for downstream statistical analysis.

### 2.13 Isolation of Mitochondria

Mitochondria were isolated from human BSM cells and murine BSM tissue using the Mitochondria Isolation Kit (Miltenyi Biotec, Auburn, CA) according to the manufacturer's instructions. In this protocol, mitochondria were isolated using immunomagnetic separation as previously described [33]. The immunomagnetic separation approach uses superparamagnetic microbeads conjugated to an antibody against the mitochondrial outer membrane protein TOM22. Briefly, 1.5 × 10^7^ cells were harvested, washed with PBS, and resuspended in lysis buffer. The cells were homogenized using a Dounce homogenizer. Homogenates were centrifuged to remove nuclei and cell debris. The resulting supernatant was centrifuged at 20 000 *g* for 15 min to obtain the mitochondrial pellet. The mitochondrial fraction was incubated with anti‐TOM22 antibody‐coated magnetic microbeads for 1 h at 4°C with gentle agitation. Using columns, magnetically labeled mitochondria were separated, washed with separation buffer, and eluted from the column.

### 2.14 Measurement of Hydrogen Peroxide Levels in Isolated Mitochondria

Mitochondrial ROS levels in isolated mitochondria were assessed using the Amplex Red Assay Kit (Invitrogen, USA) as described in [34]. Amplex Red is a fluorescent probe widely used to detect and quantify hydrogen peroxide, with detection based on the peroxide‐catalyzed oxidation of Amplex Red to resorufin. A total of 100 μL of reaction buffer (120 mM KCl, 3 mM HEPES free acid, 1 mM EGTA, and 0.3% BSA, pH 7.2, at 37°C), including 50 μM Amplex Red (Molecular Probes, Invitrogen), 6 U/mL HRP, and 25 U/mL SOD, was added for 10 min. Then, the reaction was initiated by adding 50 μg of mitochondrial proteins to 100 μL of reaction buffer. After 30 min, the absorbance of the reaction mixture was measured at 560 nm.

### 2.15 Total Cellular ATP Measurement

Total cellular ATP was measured using the CellTiter‐Glo Luminescent Assay kit (Promega, Madison, WI) according to the manufacturer's instructions. 36 h post‐infection of GFP, desmin, and vimentin in human BSM cells or in BSM strips, the growth medium was removed and replaced with 100 μL of CellTiter‐Glo assay reagent. The plate was incubated in the dark for 2 min, then gently shaken at room temperature for 10 min. After incubation, luminescence was measured using a microplate reader (BioTek, USA). ATP levels were normalized to total cell number and total protein, and relative cellular ATP levels were calculated.

### 2.16 Measurement of Mitochondrial Respiration in Intact Cells

To assess the oxygen consumption rate (OCR), an indicator of mitochondrial respiration, we used the Seahorse XFe24 extracellular flux analyzer (Agilent Technologies, Santa Clara, CA) with sequential injection of oligomycin, FCCP, and antimycin A. Human BSM cells were seeded in XF 24‐well plates. Twenty‐four hours later, cells were transduced with adenovirus encoding GFP, desmin, or vimentin and incubated for 36 h. XF24 sensor cartridges were hydrated overnight at 37°C in XE Calibrant. After 36 h of cell transduction, fresh XF Cell Mito Stress Test Assay Medium (DMEM supplemented with 5.5 mM glucose, 4 mM glutamine, and 250 μM BSA, pH 7.4) was prepared and added to the wells 1 h before the assay. Cells were then incubated at 37°C in a non‐CO_2_ incubator. Stock solutions of oligomycin, FCCP, and antimycin A were prepared in DMSO and diluted in assay medium to maintain a final DMSO concentration below 0.1%. These were loaded into the Seahorse cartridge ports to achieve final injected concentrations of 1 μM oligomycin, 0.25 μM FCCP, and 1 μM antimycin A. OCR was measured and quantified using the Seahorse analysis algorithm, with values normalized to the total cell count in each well.

### 2.17 Magnetic Twisting Cytometry

We applied spontaneous and forced motions to individual RGD‐coated ferrimagnetic microbeads (approximately 4.5 μm in diameter) anchored to the cytoskeleton via cell‐surface integrin receptors and quantified the remodeling dynamics of the CSK network and cell stiffness, respectively, using optical magnetic twisting cytometry (OMTC) [35, 36]. In brief, human BSM cells were seeded at a density of 30 000 cells/cm^2^ on collagen‐coated plastic wells (96‐well Removawell, Immulon II, Dynetech) and incubated for 24 h at 37°C in a humidified incubator containing 5% CO_2_. Adherent cells were then incubated with RGD‐coated microbeads for 20 min, followed by 3× washing in Tyrode's buffer. For an individual RGD‐coated microbead on the BSM cells, we first recorded its position every 83 milliseconds (at a frequency of 12 frames/s for *t*_max_ ~300 s) and computed the mean square displacement (MSD) of all beads as a function of time [MSD (*t*)] (nm^2^) as described [35, 37]. To study dynamic changes in cell stiffness in response to membrane depolarization, we magnetized the beads and subjected them to sinusoidal twisting (0.75 Hz), and the induced lateral bead displacements were detected optically with a spatial resolution of ~5 nm [36]. Here, the ratio of specific torque to bead displacement was calculated and expressed as the cell stiffness in pascals (Pa) per nanometer (nm). For each BSM cell, baseline stiffness was measured for the first 60 s, and dynamic changes in cell stiffness in response to 120 mM KCl were measured continuously for the next 240 s.

### 2.18 Collagen Gel Contraction Assay

BSM cell contractility was assessed using a collagen gel contraction assay, which evaluates the contraction of cells embedded in collagen gel matrices by measuring the gel size. Human BSM cells overexpressing desmin or vimentin were harvested and resuspended in a mixture of collagen I and SmGM‐2 medium with 1% FBS. The BSM cell expressing GFP served as a control. Cells at a density of 200 000 cells/ml were suspended in SmGM‐2 medium containing 1% FBS and neutralized rat tail type I collagen (Advanced Biomatrix, Carlsbad, CA) to a final concentration of 1.2 mg/mL. This mixture was seeded into a 48‐well plate and incubated at 37°C with 5% CO_2_ for 1 h to allow polymerization, then SmGM‐2 medium containing 1% FBS was added. Cells in the collagen gel were stimulated with 120 mM KCl in Tyrode buffer for 10 min, and images were acquired. A Tyrode buffer without KCl served as the vehicle. The contraction was quantified using Fiji (ImageJ/NIH), and the calculated change in gel size was used as an indicator of BSM contraction.

### 2.19 Microarray Analysis

Total RNA was isolated from human BSM cells overexpressing GFP, desmin, or vimentin using a TRIzol Plus RNA Purification System as per the manufacturer's directions. An Agilent Bioanalyzer with an RNA 6000 Nanochip was used to assess RNA quality. Microarray experiments were performed on RNA using HuGene 1.0 ST (Affymetrix) (Affymetrix, Santa Clara, CA) at the Cancer Genomics Center of Thomas Jefferson University, Philadelphia. The chips were scanned after hybridization using the Affymetrix bead array scanner. Affymetrix microarray data were analyzed by Genome Studio 3.0 and GeneSpring 14.4 software (Agilent Technologies, CA). Differentially expressed transcripts between GFP and desmin, and between GFP and vimentin, were identified using GeneSpring GX 14.4 with a *p*‐value ≤ 0.05. Differentially expressed genes were chosen as candidates for further studies based on the statistical significance of *p* ≤ 0.05 using the Student's *t*‐test (unpaired) and subsequently limited by an absolute fold change of 1.8 or more. We used a 1.8‐fold or greater change as the threshold to identify differentially expressed transcripts between control and desmin‐overexpressing BSM cells, and between control and vimentin‐overexpressing BSM cells. The probe sets were grouped by expression patterns using a hierarchical clustering algorithm, and heat maps were generated from the list of differentially expressed transcripts. The microarray dataset has been deposited in the Gene Expression Omnibus (GEO) database at the National Center for Biotechnology Information (NCBI) (https://www.ncbi.nlm.nih.gov/geo/) under accession GSE127725.

### 2.20 Cytokine Measurement by ELISA

Meso Scale Discovery's products are based on MULTI‐ARRAY technology, a proprietary combination of electrochemiluminescence detection and patterned arrays. Multiplex ELISA was performed on 4 separate cytokine panels of Meso Scale Discovery (Rockville, MD), following the manufacturer's instructions. Briefly, cell culture supernatants from GFP‐, desmin‐, or vimentin‐overexpressing human BSM cells were diluted 1:1 in 25 μL of dilution buffer provided with the Meso Scale Discovery kit and incubated with the labeled cytokine antibodies for 2 h at room temperature. After washing, the absorbance was measured using a Meso Scale Discovery luminometer.

### 2.21 Co‐Immunoprecipitation

The interaction between G0S2 and VDAC in human BSM cells was assessed by co‐immunoprecipitation, as previously described [38]. Protein samples were prepared by lysing the human BSM cells in lysis buffer (50 mM HEPES (pH 7.6), 150 mM NaCl, 1 mM EDTA, 1 mM EGTA, 10 mM MgCl_2_, 1% sodium deoxycholate, 1% Triton X‐100, protease inhibitor cocktail (1:100 dilution; Sigma, St. Louis, MO), phosphatase inhibitor cocktail (1:100 dilution; Sigma, St. Louis, MO), 0.1 mM sodium orthovanadate). The lysate was centrifuged at 20 000 × *g* for 20 min at 4°C, and the supernatant was collected. To reduce nonspecific binding to the beads, the supernatant was pre‐cleared by incubating with Dynabeads Protein A for 30 min on a rocker at 4°C. The Dynabeads were then removed from the supernatant using a magnet, and the pre‐cleared supernatant was used for subsequent immunoprecipitation. The supernatant was incubated at 4°C overnight with an anti‐G0S2 antibody. The antibody–antigen complex was captured by incubating the mixture with Dynabeads Protein A beads (Thermo Fisher Scientific, Waltham, MA) for 2 h at 4°C, followed by magnetic separation and washing. Bound proteins were eluted in sample buffer, and the beads were removed by magnetic separation before electrophoresis. The protein samples were immunoblotted with anti‐G0S2 and anti‐VDAC antibodies.

### 2.22 Animals

Transgenic mice deficient in G0S2 were kindly provided by Jose G. Teodoro (Goodman Cancer Research Center, McGill University, Canada) and bred at Thomas Jefferson University. Generation of G0S2^−/−^ mice has been described in detail elsewhere [39]. The experiments were conducted in age‐matched male G0S2 knockout (KO) mice and wild‐type (WT) controls. All experiments were conducted in accordance with the animal experimentation ethics committee guidelines of the Institute Animal Care and Use Committee at Thomas Jefferson University. The animals were maintained on a 12/12‐h light/dark cycle and supplied with food and water ad libitum. Mice were euthanized by CO_2_ asphyxiation, and death was assured by exsanguination.

### 2.23 BSM Tissue Preparation and Adenovirus Transduction

BSM tissue preparations and transduction with adenovirus were performed as described previously [16]. Briefly, BSM from WT and G0S2 KO mice was prepared by removing the serosa and urothelium, cutting them into longitudinal strips, and placing them in ice‐cold Tyrode buffer. The muscle strips were prepared for adenovirus transduction as described previously [16, 40]. Briefly, the strips were mounted onto polymerized silicone elastomer (Dow Corning, Midland, MI, USA) and they were maintained in a serum‐free medium composed of DMEM/F‐12 (1:1 ratio, Cellgro, Mediatech, Manassas, VA, USA) supplemented with 100 U/mL penicillin, 100 μg/mL streptomycin, 250 ng/mL amphotericin B, 35 μg/mL ascorbic acid, 5 μg/mL transferrin, 3.25 ng/mL selenium, 2.85 μg/mL insulin, and 200 μg/mL glutamine and placed in a 37°C, 5% CO_2_ incubator. Muscle strips were washed with sterile ice‐cold physiological salt solution (PSS) for 10 min before and after stimulation with KCl. Murine BSM strips were subjected to 120 mM KCl stimulation for 5 min daily. Muscle strips were incubated with fresh serum‐free medium after the sterile PSS wash. The muscle strips were transduced with an adenovirus encoding GFP, desmin, or vimentin for 36 h, using 10 μL of virus (titer 10^8^ pfu) per strip in 500 μL medium.

### 2.24 Measurement of Isometric Force

Contractility measurements of murine BSM strips from WT and G0S2 KO mice overexpressing desmin or vimentin were performed as described previously [16, 41]. BSM strips were attached to a force transducer and suspended in organ baths containing Tyrode buffer. Muscle strips were stretched to a force of 1.5 g. The organ baths were continuously gassed with 95% O_2_ and 5% CO_2_, and the buffer pH was maintained at 7.4. The bladder strips were equilibrated for approximately 60 min, followed by stimulation with high potassium (120 mM KCl) Tyrode buffer (7.7 mM NaCl, 120 mM KCl, 0.4 mM NaH_2_PO_4_, 1.8 mM CaCl_2_, 0.5 mM MgCl_2_, 23.8 mM NaHCO_3_, and 5.6 mM glucose), and the change in tension was recorded. After KCl stimulation, the strips were washed thoroughly with fresh Tyrode buffer. Then the strips were stimulated with carbachol at concentrations ranging from 10^−9^ to 10^−5^ M. Isometric contractions were measured using force transducers and recorded with a bridge amplifier connected to an analog‐to‐digital converter (AD Instruments Inc., Colorado Springs, CO, USA), and the data were analyzed using LabChart 7 Software. The force measurements were normalized to the cross‐sectional area of the muscle strips (g/mm^2^).

### 2.25 Statistical Analysis

GeneSpring software version 14.4 (Agilent Technologies Inc., Santa Clara, CA) was used for microarray data analyzes. A statistical threshold of *p* < 0.05 with fold‐change ≥ 1.8 or greater was considered significant for the identification of differentially expressed genes. The probe set signals were calculated using the Iterative Plier 16 summarization algorithm; the baseline‐to‐median of all samples was used as the baseline option. For other experiments, individual data points were analyzed, and differences between control and experimental groups were assessed for statistical significance using Prism version 10.4 (GraphPad, USA), with *p < 0.05, **p < 0.01, ***p < 0.001, and ****p < 0.0001 considered significant.

## 3 Results

### 3.1 Increased Expression of Desmin and Vimentin Reduces Mitochondrial Elongation and Branching

The structural details of the desmin‐GFP and vimentin‐GFP bicistronic adenoviral vector were described in our previous publication [16]. In this study, human BSM cells were transduced with adenoviruses encoding GFP, desmin‐GFP, or vimentin‐GFP, as previously described [16]. The cells were grown for 36 h post‐transduction. Human BSM cells expressing GFP served as a control. Immunoblot analysis of total protein was performed to confirm the expression levels of desmin and vimentin. The relative intensities of the desmin and vimentin bands were normalized to GAPDH. There was a significant upregulation of desmin and vimentin protein relative to control levels (Figure 1A). Desmin and vimentin expression in BSM cells remained unchanged between passages 3 and 5 (Figure 1B–D). Furthermore, expression of smooth muscle contractile proteins, smooth muscle α‐actin (SMA), smooth muscle myosin heavy chain 11 (SMHC), and SM22α remained unchanged in BSM cells between passages 3 and 5, confirming the contractile phenotype of the cells (Figure 1B–D). Prior studies have used BSM cells up to 6 passages, demonstrating that passage maintains the contractile phenotype [42, 43, 44, 45]. We carried out TEM to assess potential changes in mitochondrial ultrastructure associated with desmin and vimentin overexpression. Morphological analysis revealed that increased expression of desmin and vimentin did not induce structural abnormalities, and mitochondrial morphology remained comparable to that of control cells (Figure 1E).

**FIGURE 1:**
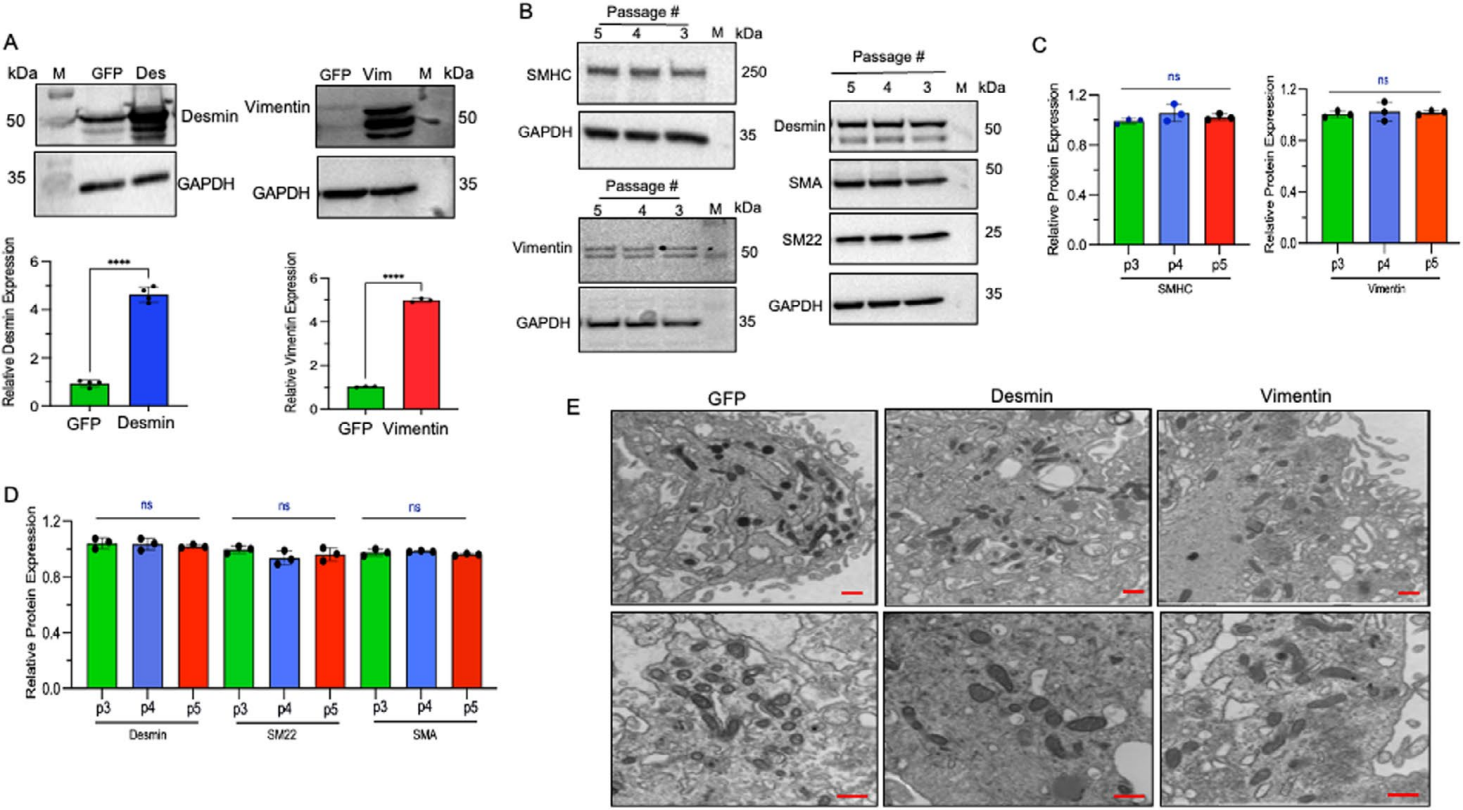
Increased expression of desmin and vimentin does not cause mitochondrial structural abnormalities. (A) Human BSM cells were transduced with an adenovirus encoding desmin and vimentin, and GFP for 36 h. The expression levels of desmin and vimentin proteins were analyzed by immunoblotting following their overexpression. GAPDH served as a loading control. Representative immunoblots and corresponding quantifications are shown. (B–D) The expression levels of contractile proteins and desmin and vimentin proteins across passages (P3–P5) were analyzed by immunoblotting. GAPDH served as a loading control. Representative immunoblots and their corresponding quantifications are shown in panels B, C, and D. (E) *TEM imaging*: Human BSM cells overexpressing desmin and vimentin were fixed and processed for TEM. GFP‐expressing cells were used as a control group. The TEM images in panel E depict mitochondria from lower (upper panel) and higher (lower panel) magnifications. Scale bars = 500 nm. *M* denotes molecular weight marker. Data are presented as mean ± SD; *n* = 3–4 in A, *n* = 3 in B–D, and *n* = 2 in E. The ns indicates non‐significant. ****p < 0.0001.

We have used live‐cell confocal microscopy to characterize mitochondrial shapes. We determined the mitochondrial aspect ratio (AR) and form factor (FF) as indicators of mitochondrial shape in human BSM cells overexpressing desmin and vimentin. An analysis using a Python‐based pipeline to extract shape metrics from confocal fluorescence microscopy images revealed that desmin‐ and vimentin‐overexpressing cells display lower AR and FF values than control cells (Figure 2A–C), indicating reduced mitochondrial elongation. Human BSM cells overexpressing desmin and vimentin exhibit decreased total and mean branch length compared to control cells (Figure 2D,E). Although the number of branches remains unchanged (Figure 2F), the shorter branch lengths suggest a more disconnected and simplified network structure.

**FIGURE 2:**
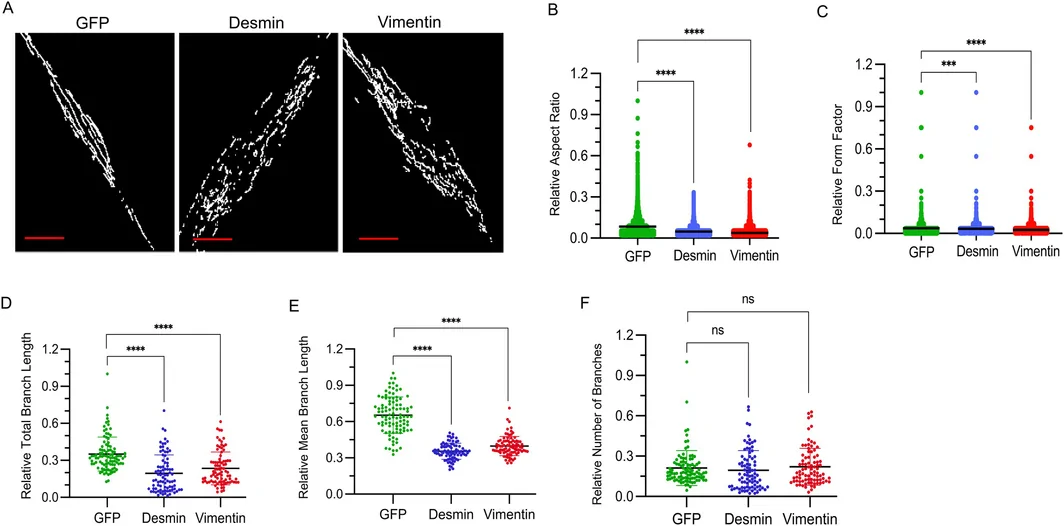
Increased expression of desmin and vimentin reduces mitochondrial elongation and branching. (A) Human BSM cells expressing DsRed2‐Mito‐7 were transduced with adenovirus encoding GFP, desmin, and vimentin for 36 h, and the mitochondria in these cells were visualized via confocal microscopy. GFP‐expressing cells served as the control group. Confocal images of mitochondria were processed using Python‐based pipelines, as described in the Materials and Methods section. Representative confocal images are shown in panel A. The white color in panel A represents mitochondria. (B–F) Quantification of mitochondrial length and circularity, represented by aspect ratio and form factor, respectively. The quantification of the branching parameter is shown in panels D–F. Scale bars = 50 μm. Data are presented as mean ± SD; *n* > 75 cells per group. Ns indicates non‐significant. ***p < 0.001 and ****p < 0.0001.

### 3.2 Increased Expression of Desmin and Vimentin Reduces Mitochondrial Fusion Proteins

The expression levels of proteins involved in mitochondrial dynamics, including fusion and fission proteins, were assessed by immunoblotting. There was a significant downregulation of MFN1 and MFN2 in desmin‐ and vimentin‐overexpressing human BSM cells compared to control cells (Figure 3A–C). There was a significant increase in Fis1 protein, which is involved in mitochondrial fission, along with elevated levels of phospho‐DRP1 (Ser616), in desmin‐ and vimentin‐overexpressing BSM cells. However, there were no significant differences in DRP1 or OPA1 expression levels between the control and desmin‐overexpression or between the control and vimentin‐overexpression.

**FIGURE 3:**
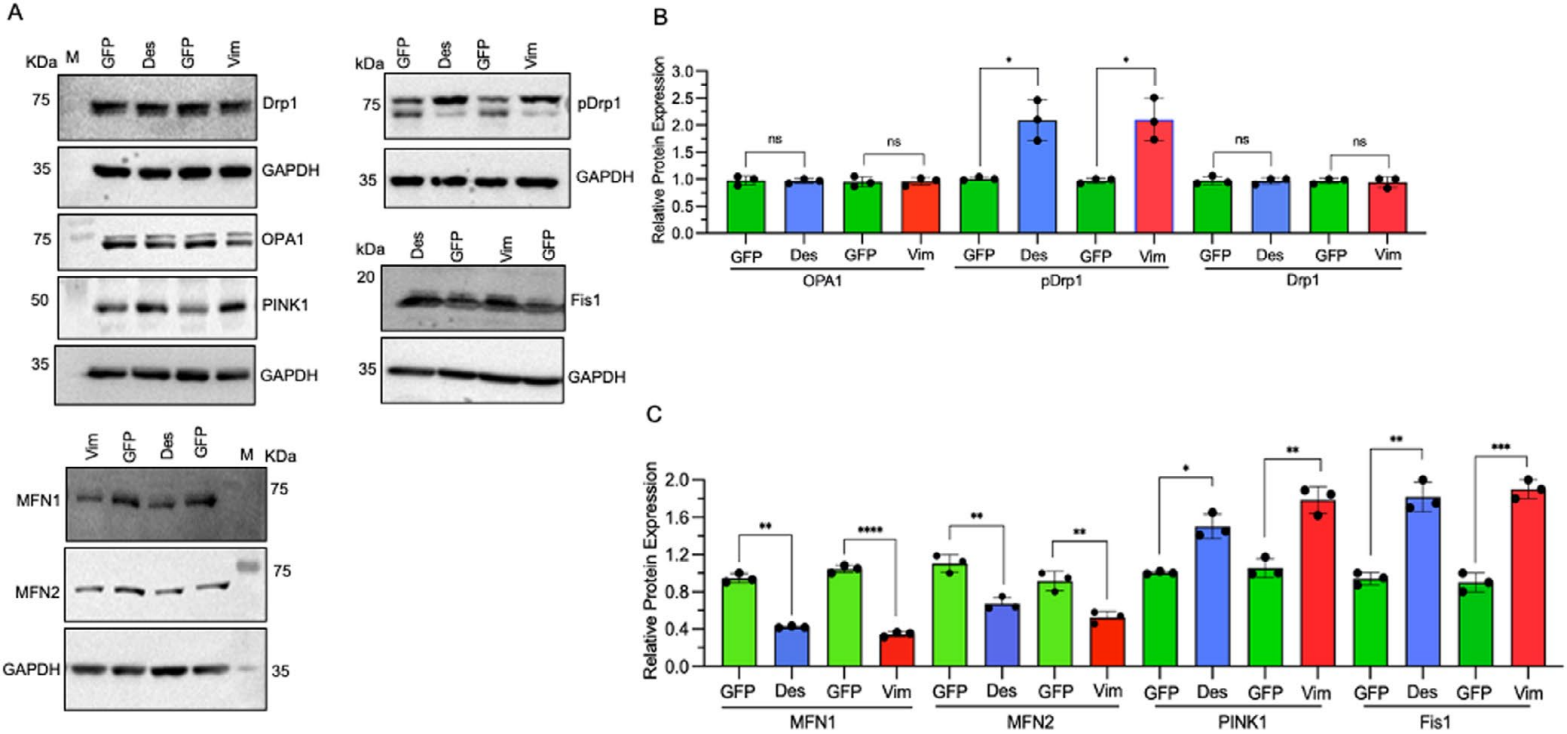
Increased expression of desmin and vimentin reduces the levels of the mitochondrial fusion proteins MFN1 and MFN2. Human BSM cells were transduced with an adenovirus encoding desmin and vimentin, and GFP for 36 h. Human BSM cells overexpressing desmin and vimentin were processed for immunoblot. GFP‐expressing cells served as a control group. The protein expression levels of OPA1, DRP1, Fis1, PINK1, MFN1, and MFN2 were analyzed by immunoblotting. Additionally, the phospho‐DRP1 level was determined. GAPDH served as the loading control. Representative immunoblots and their corresponding quantifications are shown in panels A–C. Data are presented as mean ± SD, *n* = 3. The ns indicates non‐significant. *p < 0.05, **p < 0.01, ***p < 0.001 and ****p < 0.0001.

### 3.3 Increased Expression of Desmin and Vimentin Increases Mitochondrial ROS and Reduces OCR and ATP Levels

To quantify mitochondrial ROS levels, live cells were stained with MitoSOX Red and co‐localized with MitoTracker Deep Red to ensure that ROS measurements were specific to mitochondria. Cells were imaged using the Nikon A1R. Live‐cell imaging revealed that overexpression of desmin or vimentin increased MitoSOX Red fluorescence intensity relative to control cells, indicating elevated mitochondrial ROS levels (Figure 4A,B). Additionally, ROS levels in isolated mitochondria were measured using the Amplex Red Hydrogen Peroxide/Peroxidase assay. Consistent with live‐cell imaging, overexpression of desmin and vimentin resulted in elevated ROS levels in isolated mitochondria relative to GFP‐expressing cells (Figure 4C). Elevated expression of desmin and vimentin leads to decreased basal and maximal OCR (Figure 4D) and total ATP levels (Figure 4E) in human BSM cells.

**FIGURE 4:**
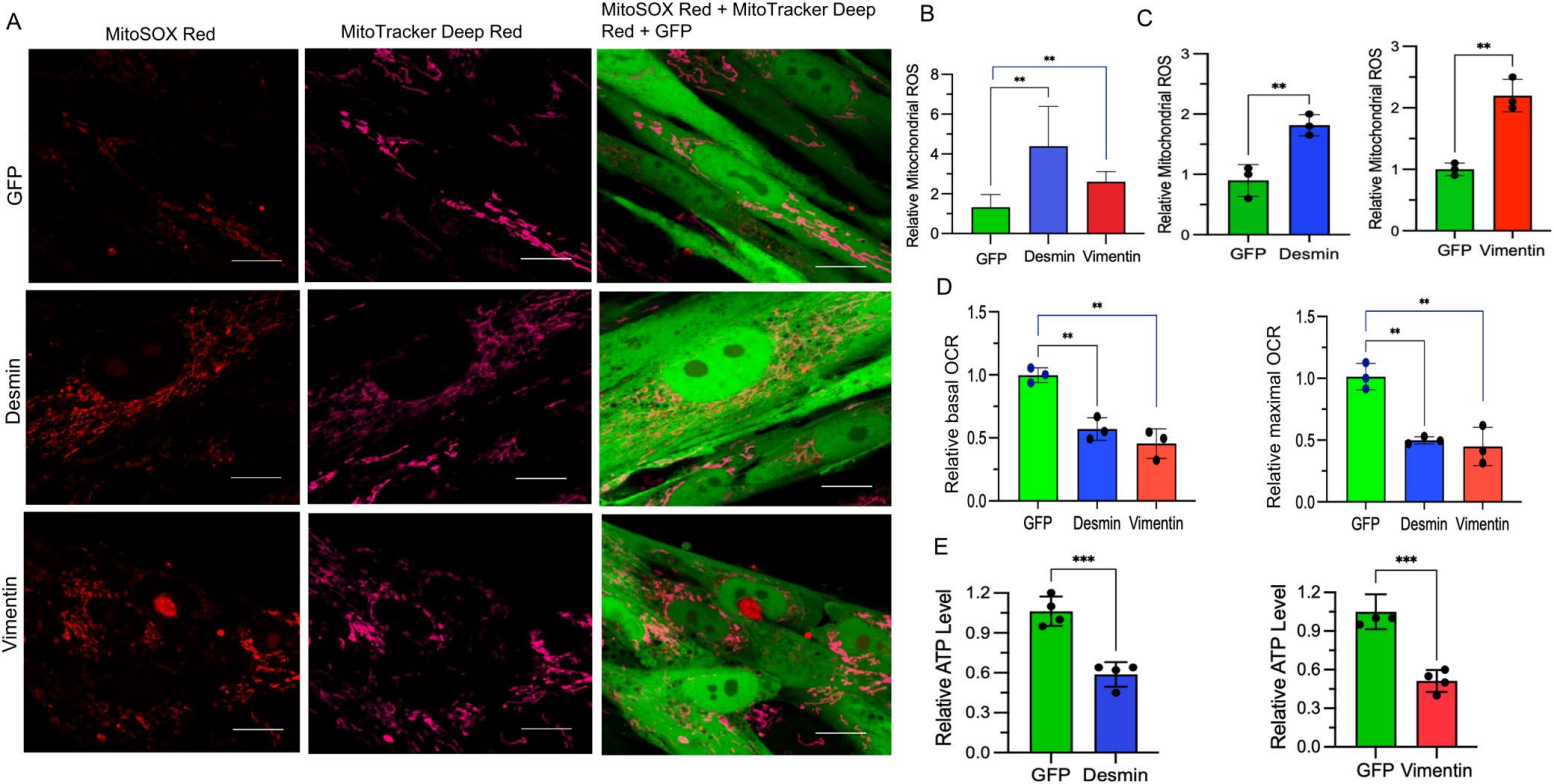
Increased expression of desmin and vimentin enhances mitochondrial ROS and reduces oxygen consumption rate and ATP levels. Human BSM cells were transduced with an adenovirus encoding desmin and vimentin, and GFP for 36 h. Human BSM cells overexpressing desmin and vimentin were processed for ROS, OCR, and ATP measurements. GFP‐expressing cells served as a control group. (A) Mitochondrial ROS levels in live cells were assessed by MitoSOX fluorescence. Human BSM cells overexpressing desmin and vimentin were stained with MitoSOX (5 μM) and MitoTracker DeepRed (10 nM), then visualized by confocal microscopy. Images were captured using a 100× oil‐immersion objective on a Nikon AIR confocal microscope. Mitochondrial ROS levels were measured by fluorescence intensity of MitoSOX Red. GFP‐expressing human BSM cells were used as a control group. Scale bars = 50 μm. (B) Quantifications of MitoSOX fluorescent intensity. Live cell confocal images of mitochondria were processed using Python‐based pipelines, as described in the Materials and Methods section. (C) ROS levels in isolated mitochondria were assessed by measuring H_2_O_2_ production using the Amplex Red Hydrogen Peroxide assay in the presence of superoxide dismutase. (D) OCR was measured using a Seahorse Bioscience extracellular flux analyzer. Basal and maximal respiration were determined. OCR values were normalized to the total number of cells. (E) Quantifications of total cellular ATP levels by a bioluminescence assay. ATP levels were measured based on firefly luciferin‐luciferase luminance intensity. Data are presented as mean ± SD, and *n* = 25 cells in each group in B, *n* = 3 in C and D, and *n* = 4 in E. **p < 0.01 and ***p < 0.001.

### 3.4 Increased Expression of Desmin and Vimentin Reduced the Cell Stiffness and Contraction in Human BSM Cells

Force generation in smooth muscle is not driven solely by the myosin‐actin sliding mechanism; it also relies on the cytoskeletal network, which provides structural support and anchoring, thereby transmitting force efficiently to the surrounding tissue. Reorganization of intermediate filaments modulates contractility during stimulation, and disruption of this process compromises the muscle's contractile properties [46]. To probe the dynamics of cytoskeletal remodeling in human BSM cells overexpressing desmin and vimentin, we measured the spontaneous motion of an individual RGD‐coated microbead anchored to the cytoskeleton. Increased expression of desmin and vimentin led to a significant increase in the mean squared displacement (MSD), indicative of cytoskeletal remodeling (Figure 5A); the increase was more pronounced in vimentin‐overexpressing cells than in desmin‐overexpressing cells. Consistent with these findings, vimentin overexpressing cells exhibited a significant decrease in the cell stiffness (Figure 5B); there were no statistical differences in basal stiffness between desmin overexpressing cells and GFP control cells (Figure 5B). Strikingly, compared to GFP‐expressing cells, vimentin overexpressing cells showed a marked attenuation in KCl‐induced cell stiffening responses (Figure 5C); in contrast, desmin overexpressing cells did not exhibit a significant reduction in cell stiffness compared to control cells following KCl stimulation (Figure 5C).

**FIGURE 5:**
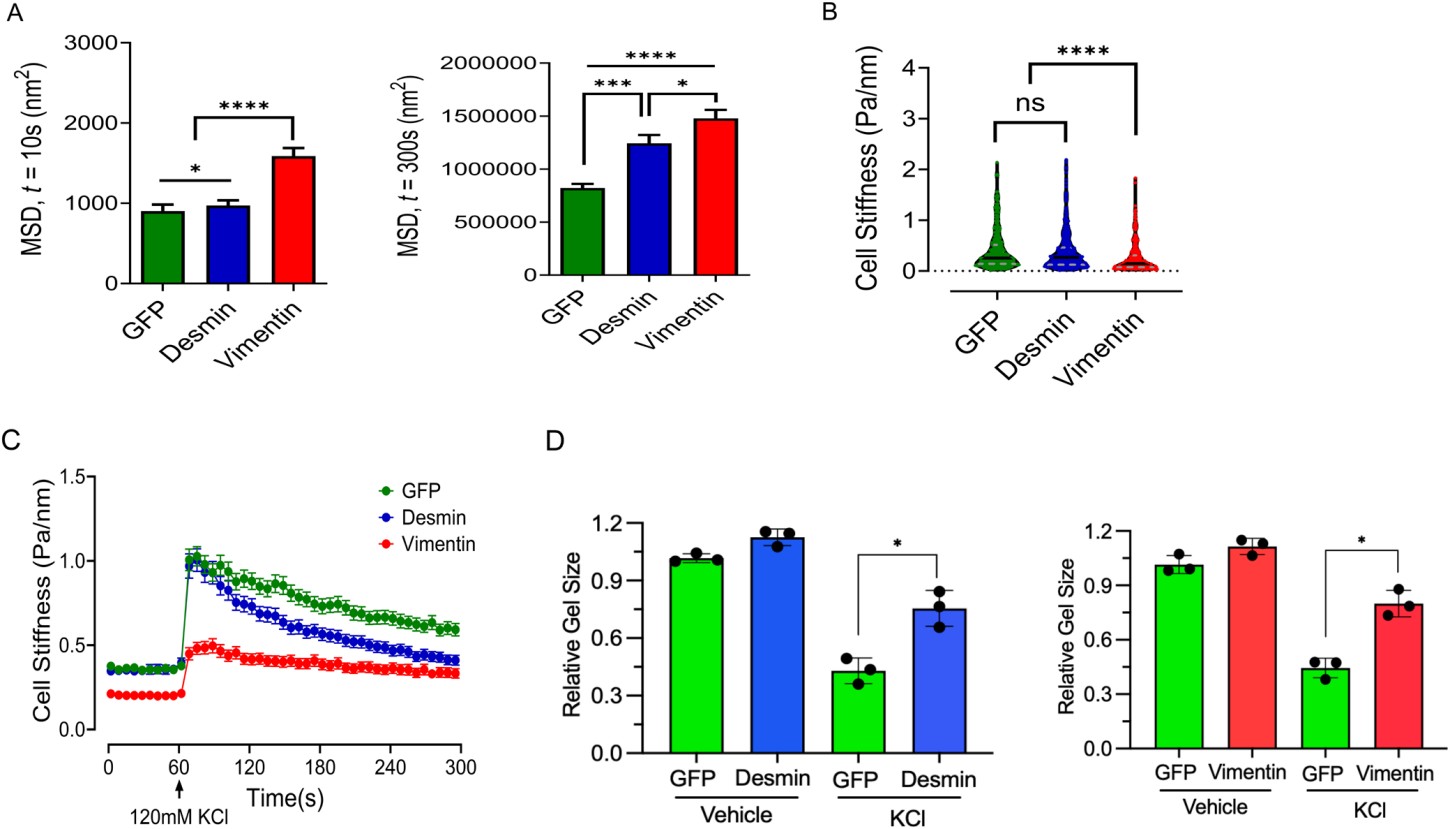
Desmin and vimentin overexpression reduce cell stiffness and contraction. Human BSM cells overexpressing desmin and vimentin proteins were processed for MTC as described in Materials and Methods. GFP‐expressing cells were used as a control group. (A) Spontaneous bead motions are quantified by their mean squared displacement (MSD) as a function of time. The MSDs were computed at equally spaced time intervals (1.3 s). Cytoskeletal remodeling is inferred from the increase in MSD over time. (B, C) Stiffness was measured continuously for 300 s for each BSM cell overexpressing desmin and vimentin, along with control and vehicle‐treated cells: Dynamic changes in cell stiffness in response to 120 mM KCl were measured continuously for 240 s, as an indication of single‐cell contraction. Data are recorded at equally spaced time intervals (1.3 s), but for clarity, are suppressed at 3.9 s intervals. (D) For gel contraction studies, human BSM cells overexpressing desmin and vimentin were seeded in a collagen gel as described in Section 2, and the cells in collagen gels were subjected to KCl (120 mM) stimulation. Subsequently, the gels were imaged. The collagen gel size in each image was quantified using ImageJ (NIH) software, and the percentage change from baseline in response to membrane depolarization was calculated. Data are presented as mean ± SD (*n* = 279–348 cells) in A–C and *n* = 3 in D. NS indicates non‐significant. *p < 0.05, ***p < 0.001 and ****p < 0.0001.

We next measured the contraction of BSM cells overexpressing desmin and vimentin in response to KCl stimulation using a collagen gel contraction assay, in which gel size is inversely proportional to the extent of cell contraction. GFP‐expressing BSM cells showed a marked decrease in gel size in response to 120 mM KCl (Figure 5D), indicating significant contraction. In contrast, overexpression of desmin or vimentin resulted in increased gel size compared to GFP‐expressing cells following KCl stimulation, reflecting reduced contractile responses (Figure 5D). Collectively, these findings suggest that increased expression of desmin and vimentin attenuates depolarization‐induced contraction in human BSM cells. Human BSM cells overexpressing desmin or vimentin were examined by TEM to assess changes in smooth muscle actin and myosin filaments. Increased expression of desmin or vimentin did not induce any gross changes in actin and myosin filaments (Figure 6).

**FIGURE 6:**
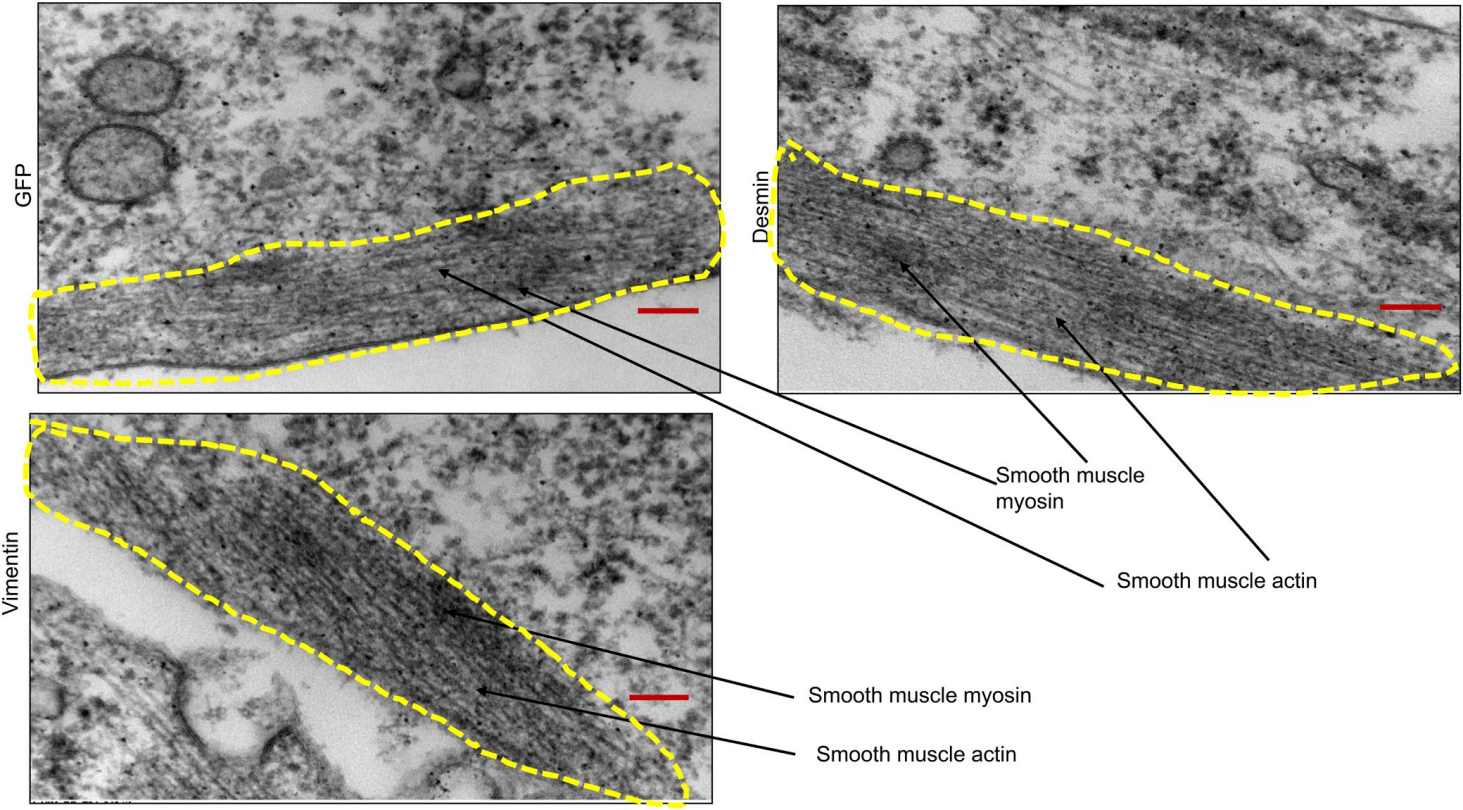
Increased expression of desmin and vimentin did not induce obvious changes in actin and myosin filaments. Human BSM cells overexpressing desmin and vimentin were processed for TEM as described in the Materials and Methods section. Briefly, cells were fixed in 2.5% glutaraldehyde in 0.1 M sodium cacodylate buffer (pH 7.4) and then post‐fixed in 2% osmium tetroxide (OsO_4_) in 0.1 M sodium cacodylate buffer (pH 7.4). Fixed cells were dehydrated through a graded series of acetone and embedded in Spurr resin. GFP‐expressing cells were used as a control group. Smooth muscle actin and smooth muscle myosin filaments are indicated. We have analyzed 15 TEM images from each group. Scale bar = 100 nm.

### 3.5 Localization of Desmin and Vimentin Filaments in Human BSM Cells Overexpressing Desmin or Vimentin

Human BSM cells overexpressing desmin or vimentin were stained with desmin‐ or vimentin‐specific antibodies and examined by confocal microscopy. In control cells and in cells overexpressing desmin or vimentin, both desmin and vimentin form filaments throughout the cytoplasm (Figures 7A and 8A). Confocal images showed that increased expression of desmin and vimentin did not alter filament localization, which remained consistent with the pattern observed in control BSM cells (Figures 7A and 8A). The filaments form an intricate network of branched and overlapping filaments. In some regions, in both control and desmin‐ and vimentin‐overexpressing cells, thick, intense filamentous structures are observed. Consistent with our findings, previous studies have reported that desmin and vimentin form an intricate network of branched and overlapping filaments and thick, intense filamentous structures in rat aortic smooth muscle [47] and in the smooth muscle of murine bladder tissues [16]. In this study, Python‐based skeletonization of high‐resolution confocal images revealed that the desmin and vimentin filaments in human BSM cells form a highly interconnected, branching network (Figures 7B and 8B). As a result, we could not reliably resolve individual filaments or estimate their filament distribution. Qualitatively, desmin and vimentin filaments showed no significant differences in filament pattern between control and desmin‐overexpressing conditions, or between control and vimentin‐overexpressing conditions (Figures 7A,B and 8A,B).

**FIGURE 7:**
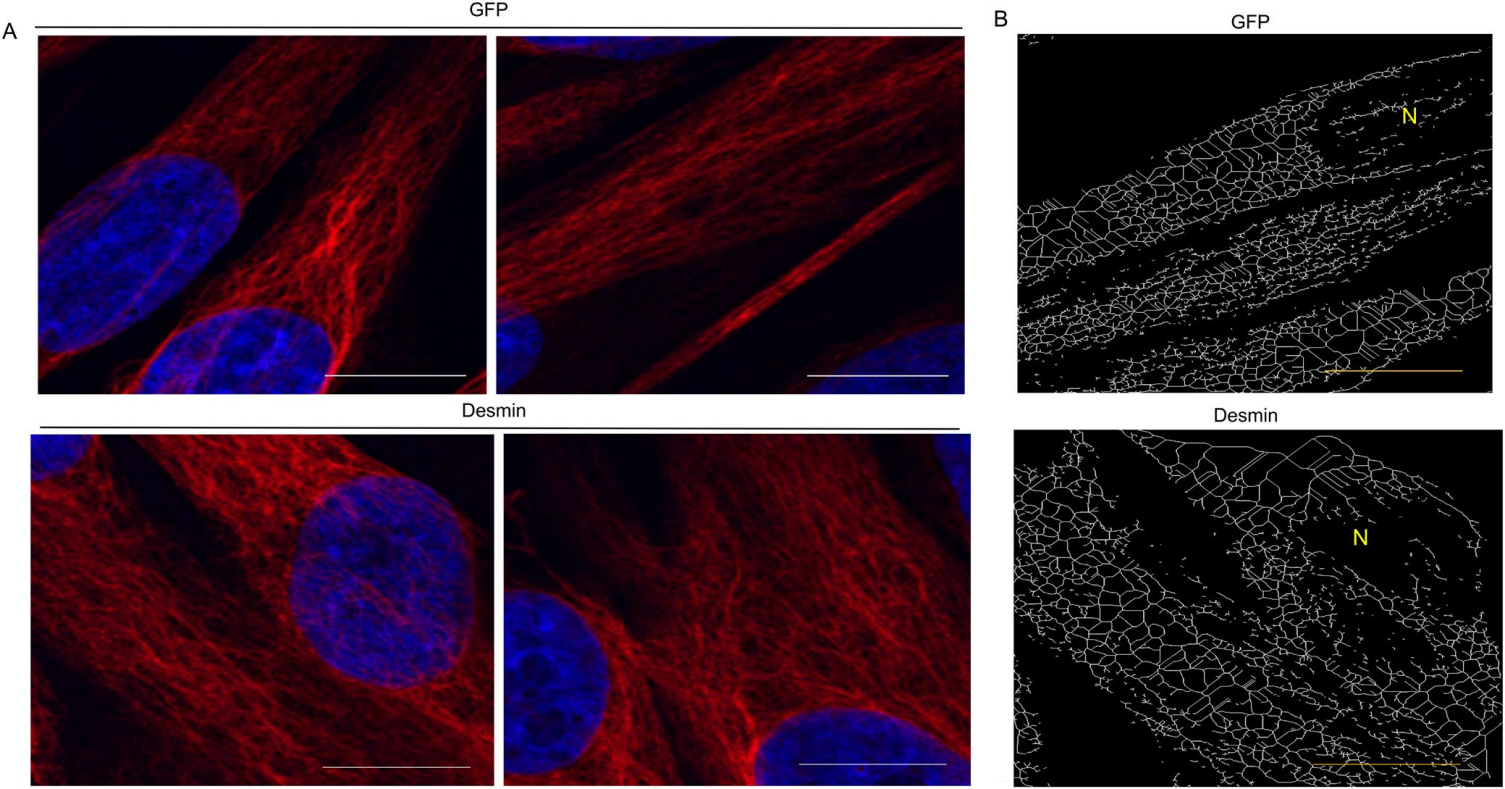
Localization of desmin filaments in human BSM cells overexpressing desmin. (A, B) Human BSM cells were transduced with an adenovirus encoding desmin for 36 h. BSM cells overexpressing desmin were immuno‐stained after incubation with Alexa Fluor 555‐labeled desmin antibody. GFP‐expressing cells served as the control group. Desmin filaments were imaged by confocal microscopy. Confocal images were acquired using identical microscope settings, with autoscaling applied across groups. Auto‐scaled images were used for visualization and to determine the subcellular distribution of filaments. (A) Representative confocal images are shown. (B) Confocal images of desmin filaments were analyzed using a Python‐based skeletonization pipeline. Binary masks were generated and reduced to 1‐pixel‐wide skeletons to view filament organization. Skeletons were generated from segmentation inputs using a Python‐based analysis pipeline. Due to the high density and interconnected filaments, identifying individual filament lengths was not feasible. Representative images are shown in panel B. Twenty‐five confocal microscopic images were analyzed for each group. *N* denotes the nucleus. Scale bar = 10 μm.

**FIGURE 8:**
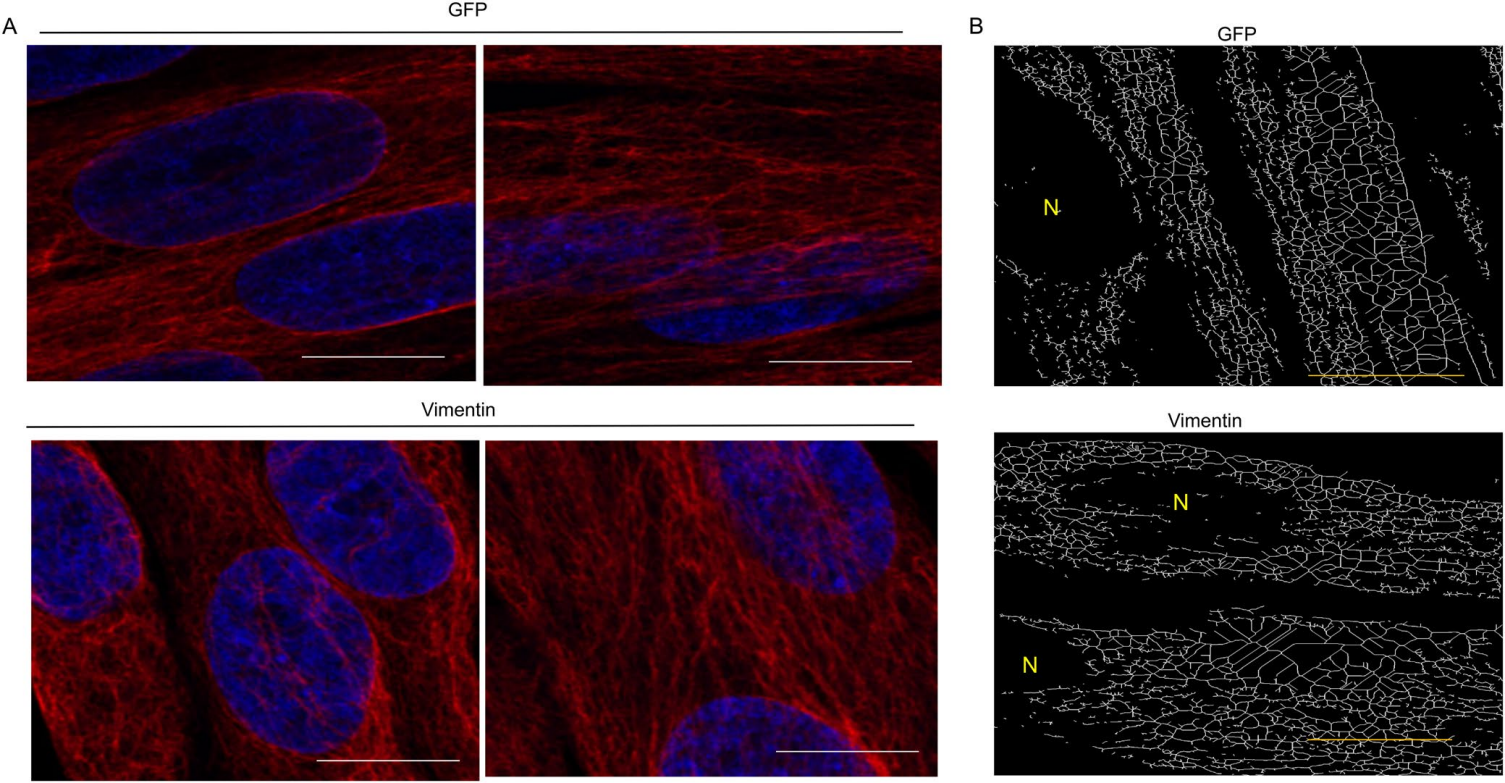
Localization of vimentin filaments in human BSM cells overexpressing vimentin. (A, B) Human BSM cells were transduced with an adenovirus encoding vimentin for 36 h. BSM cells overexpressing vimentin were immuno‐stained after incubation with Cy3‐labeled vimentin antibody. GFP‐expressing cells served as the control group. Vimentin filaments were imaged by confocal microscopy. Confocal images were acquired using identical microscope settings, with autoscaling applied across groups. Auto‐scaled images were used for visualization and to determine the subcellular distribution of filaments. (A) Representative confocal images are shown. (B) Vimentin filaments were analyzed using a Python‐based skeletonization pipeline. Binary masks were generated and reduced to 1‐pixel‐wide skeletons to view filament organization. Skeletons were generated from segmentation inputs using a Python‐based analysis pipeline. Due to the high density and interconnected filaments, identifying individual filament length was not feasible. Representative images are shown in panel B. 25 confocal microscopic images were analyzed for each group. *N* denotes the nucleus. Scale bar = 10 μm.

### 3.6 Overexpression of MnSOD Did not Rescue the Decreased Contractile Response and the Reduced ATP Levels in Human BSM Cells With Elevated Levels of Desmin and Vimentin

MnSOD (SOD2), located in the mitochondrial matrix, scavenges superoxide radicals generated by the electron transport chain. Overexpression of MnSOD did not mitigate the decreased contractile response to KCl stimulation caused by desmin and vimentin overexpression (Figure 9A). Overexpression of MnSOD did not restore mitochondrial ATP levels, although it partially reduced the elevated mitochondrial ROS caused by desmin and vimentin overexpression (Figure 9B,C). Cells treated with antimycin A for 36 h were assessed for the expression levels of desmin and vimentin. Immunoblot analysis shows that antimycin A treatment does not affect the expression of desmin or vimentin, despite significantly increasing mitochondrial ROS production (Figure 9D–F).

**FIGURE 9:**
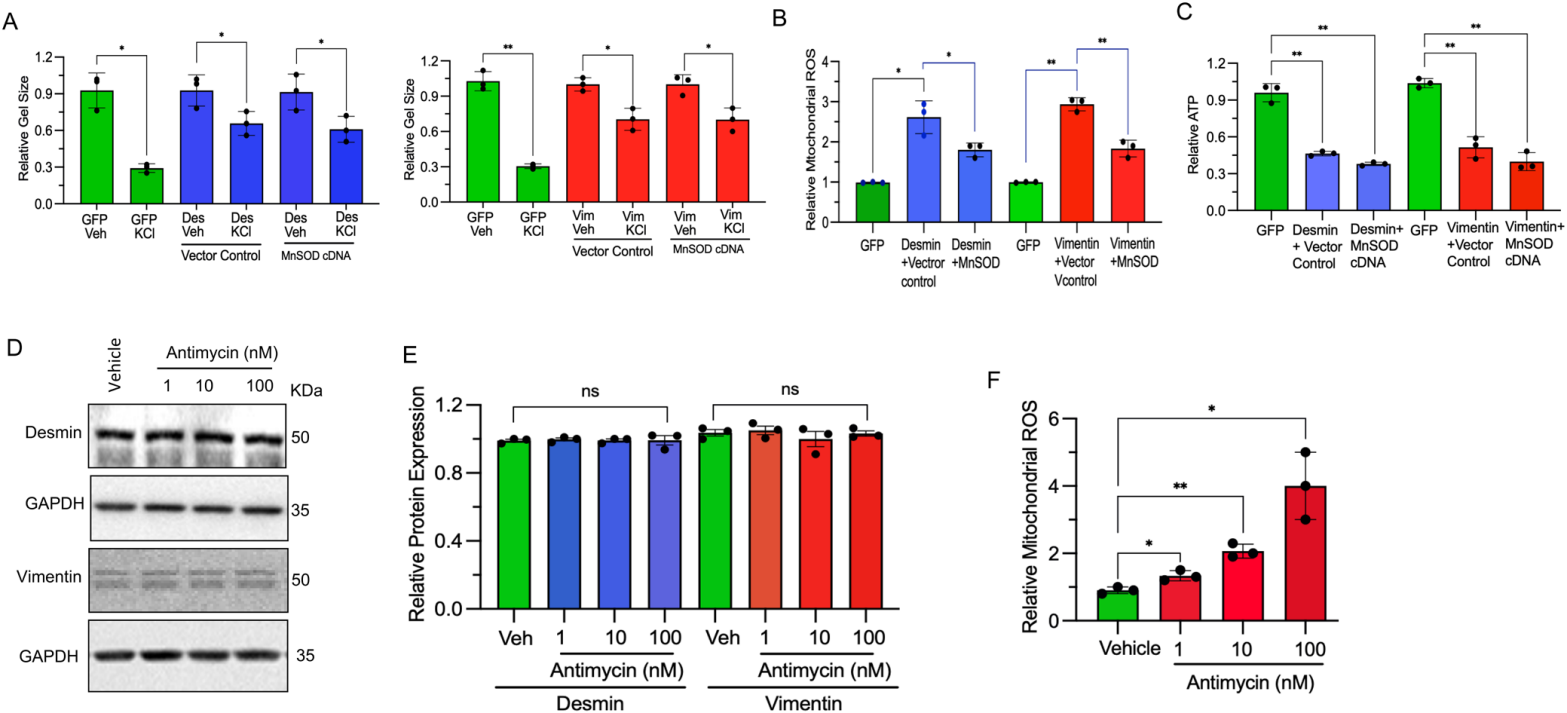
Overexpression of MnSOD did not rescue the decreased contractile response and reduced ATP levels in human BSM cells with elevated levels of desmin and vimentin. Human BSM cells were transfected with MnSOD cDNA and transduced with adenoviruses encoding desmin and vimentin. (A) *Gel contraction*: For gel contraction studies, cells were seeded in a collagen gel as described in the Materials and Methods section, and gel contraction was measured in response to 120 mM KCl stimulation. The gels were subsequently imaged. The size of collagen gel in each image was quantified using Fiji (ImageJ/NIH) software, and the percentage change was calculated. (B) *Measurement of mitochondrial ROS levels*: ROS levels in cells were measured using isolated mitochondria with the Amplex Red Hydrogen Peroxide/Peroxidase Assay Kit. (C) *Quantifications of total cellular ATP levels*: ATP levels were measured based on firefly luciferin‐luciferase luminescence intensity. (D, E) *Effect of antimycin on desmin and vimentin expression*. Human BSM cells were treated with the indicated concentrations of antimycin A for 36 h. The expression levels of desmin and vimentin proteins were analyzed by immunoblot. GAPDH served as a loading control. Representative immunoblots and their corresponding quantification are shown in D, E panels. Control cells received an equivalent volume of DMSO to vehicle treatment, and the final DMSO concentration was maintained at ≤ 0.1%. (F) *Measurement of mitochondrial ROS*: ROS levels in control and antimycin A‐treated human BSM cells were measured using isolated mitochondria with the Amplex Red Hydrogen Peroxide/Peroxidase Assay Kit. Data are presented as mean ± SD, and *n* = 3. The ns indicates non‐significant. *p < 0.05 and **p < 0.01.

### 3.7 Overexpression of Desmin and Vimentin Increases Expression of G0S2, a Mitochondrial Protein, in Human BSM Cells

We obtained a global gene expression profile in human BSM cells overexpressing either desmin or vimentin using the HuGene 1.0 ST (Affymetrix). Human BSM cells expressing GFP served as a control. All gene expression data from human BSM cells overexpressing desmin or vimentin were compiled and differentially expressed transcripts between GFP and desmin and between GFP and vimentin were identified using GeneSpring GX 14.4. The list of differentially expressed transcripts with 1.8‐fold or greater change between control and desmin‐overexpressing human BSM cells, and between control and vimentin‐overexpressing human BSM cells, is provided in Figures S1 and S2. Differential gene expression analysis identified 476 differentially expressed transcripts in human BSM cells overexpressing desmin. Among these, 432 are mapped to annotated genes, whereas 44 lack gene‐level annotation and a gene symbol. Of these, 216 genes were upregulated, and 216 genes were downregulated in human BSM cells overexpressing desmin (Figure S1). Differential gene expression analysis identified 572 differentially expressed genes in human BSM cells overexpressing vimentin. Of these, 187 genes were upregulated, and 385 genes were downregulated (Figure S2).

Hierarchical clustering of gene expression reveals overlapping patterns among control, desmin‐overexpressing, and vimentin‐overexpressing cells (Figure 10A). The analysis highlights genes that are significantly upregulated or downregulated in human BSM cells overexpressing desmin or vimentin compared with control cells (Figure 10A). Among the top‐ranking differentially expressed genes, IL‐6 and G0S2 were highly upregulated, and CAV‐1 was downregulated by desmin or vimentin (Figure 10A and Figures S1 and S2). To validate the expression changes observed by microarray analysis, we tested G0S2 and caveolin‐1 by immunoblotting and IL‐6 and IL‐1β by ELISA. The G0S2 protein expression is significantly induced in desmin‐ and vimentin‐overexpressing BSM cells (Figure 10B). Caveolin‐1 downregulation (Figure 10C) and IL‐6 and IL‐1β upregulation (Figure 10D) in human BSM cells overexpressing desmin or vimentin were confirmed by immunoblot and ELISA, respectively. Furthermore, co‐immunoprecipitation using G0S2 antibody revealed that G0S2 interacts with VDAC in human BSM cells overexpressing desmin or vimentin (Figure 10B). Immunoblot analysis of VDAC across passages P3–P5 showed no significant differences (Figure 10E). Cytochrome c oxidase IV (COX IV) served as a mitochondrial loading control (Figure 10E).

**FIGURE 10:**
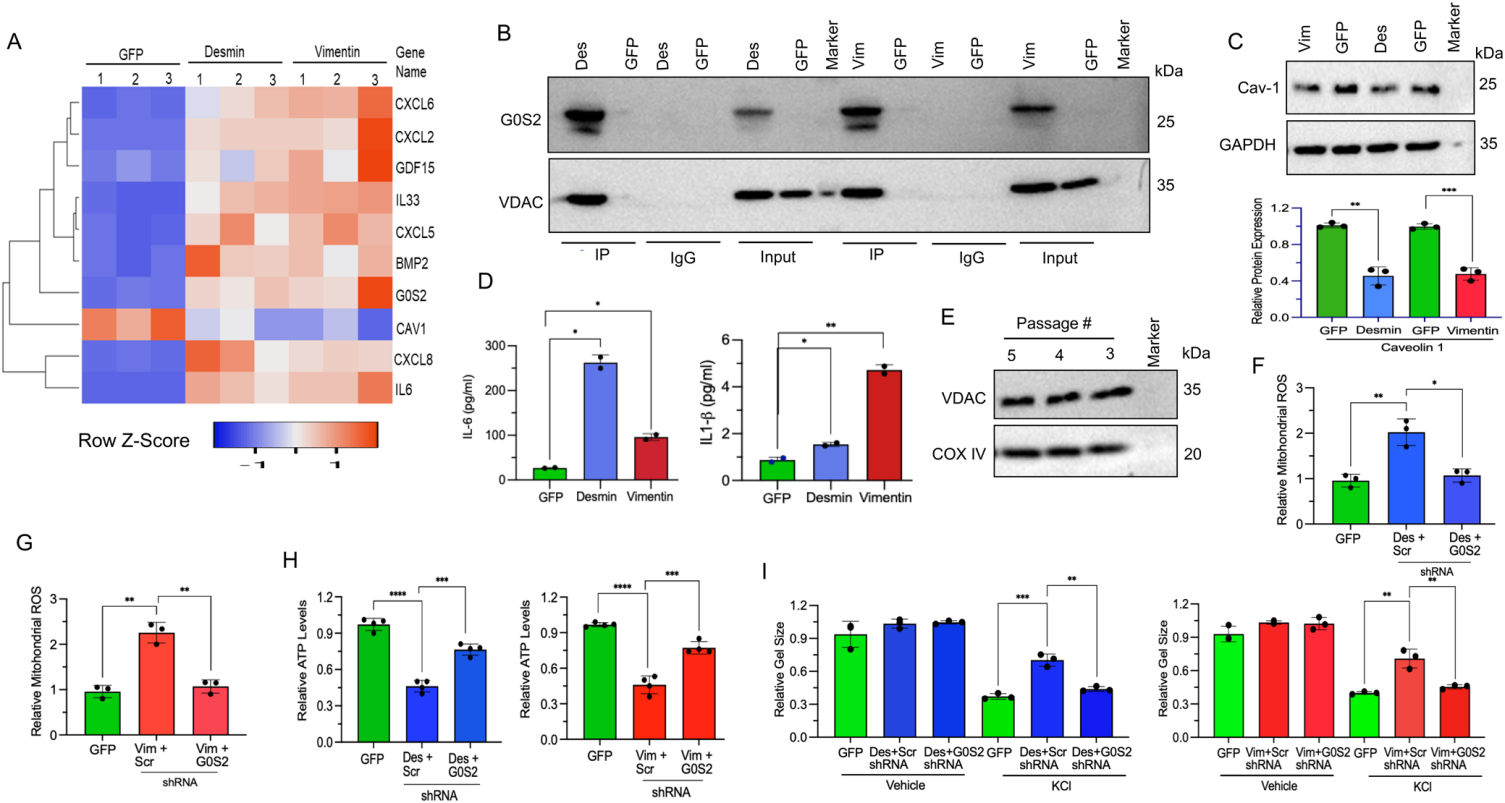
Increased expression of desmin and vimentin induces mitochondrial and contractile dysfunction via G0S2 in human BSM cells. (A) *Microarray analysis*: Gene expression was analyzed using microarray‐based transcriptomics. A heatmap generated by hierarchical clustering illustrates trends in gene expression in human BSM cells overexpressing desmin or vimentin proteins compared with GFP‐expressing control cells. Each row represents a single mRNA probe, and the accompanying dendrogram shows the hierarchical clustering tree of the gene expression data. The colored bar at the bottom represents changes in gene expression on a linear scale, with blue indicating down‐regulation and red indicating up‐regulation. (B) *G0S2 interaction with VDAC*: Mitochondrial proteins from GFP‐, desmin‐, and vimentin‐overexpressing human BSM cells were subjected to co‐immunoprecipitation. Mitochondrial proteins were immunoprecipitated using an anti‐G0S2 antibody. The resulting immunoprecipitate was separated by SDS‐PAGE and probed with anti‐G0S2 and anti‐VDAC antibodies. (C) The protein expression levels of CAV1 were analyzed by immunoblotting. Representative immunoblot and its corresponding quantification are shown in panel C. (D) The levels of IL‐6 and IL‐1β were analyzed by ELISA. (E) The expression levels of VDAC protein across passages (P3–P5) were analyzed by immunoblotting. Cytochrome c oxidase IV (COXIV) served as a loading control. (F–I) Human BSM cells were transduced with adenovirus encoding GFP, desmin (Des), and vimentin (Vim) in the presence of scrambled (Scr) shRNA or G0S2 shRNA. (F, G) ROS levels in cells were measured using isolated mitochondria with the Amplex Red Hydrogen Peroxide/Peroxidase Assay Kit. (H) *Quantification of total cellular ATP levels*. ATP levels were measured based on firefly luciferin‐luciferase luminescence intensity. (I) *BSM gel contraction*: For gel contraction studies, cells were seeded in a collagen gel as described in Materials and Methods, and gel contraction was measured in response to 120 mM KCl stimulation. The gels were subsequently imaged. The size of the collagen gel in each image was quantified using Fiji (ImageJ/NIH) software, and the percentage change was calculated. Data are presented as mean ± SD, with *n* = 3 in A, *n* = 3 in C, F, G, and I, and *n* = 2 in D, *n* = 4 in H. *p < 0.05, **p < 0.01, ***p < 0.001 and ****p < 0.0001.

To investigate the effects of G0S2 on desmin and vimentin‐overexpression‐induced mitochondrial dysfunction, we measured mitochondrial ROS and ATP levels in cells transduced with scrambled or G0S2‐targeting shRNA under conditions of desmin and vimentin overexpression. Knockdown of G0S2 restored mitochondrial ATP levels and reduced the elevated mitochondrial ROS caused by desmin and vimentin overexpression (Figure 10F–H). To investigate the effects of G0S2 on desmin and vimentin overexpression‐induced contractile dysfunction, we measured the contractile response of BSM cells overexpressing desmin and vimentin in the presence of scrambled or G0S2‐targeted shRNA in response to KCl stimulation. Knockdown of G0S2 prevented the reduction in contractile response (Figure 10I) induced by desmin and vimentin overexpression. Collectively, our results demonstrate that desmin and vimentin elicited their effect on contractility and mitochondrial function via G0S2 in human BSM cells.

### 3.8 Functional Role of G0S2 in Murine BSM Contractility and Mitochondrial Functions

Next, we determined the role of G0S2 in regulating contractile function in murine BSM tissue. Murine BSM strips from wild‐type (WT) and G0S2 KO mice were transduced with an adenoviral vector encoding desmin or vimentin for 36 h, and the contractile responses to KCl and carbachol were assessed. Desmin or vimentin overexpression reduced the peak force developed by BSM strips from WT mice in response to KCl and carbachol compared to the force developed by GFP‐expressing murine BSM strips from WT mice (Figure 11A,B). Notably, KCl‐ and carbachol‐induced contraction was not reduced in the BSM strips from G0S2 KO mice in response to increased expression of desmin or vimentin (Figure 11C,D). There was no significant difference in peak force between the BSM strips expressing GFP from WT and G0S2 KO mice in response to KCl and carbachol (Figure 11A–D).

**FIGURE 11:**
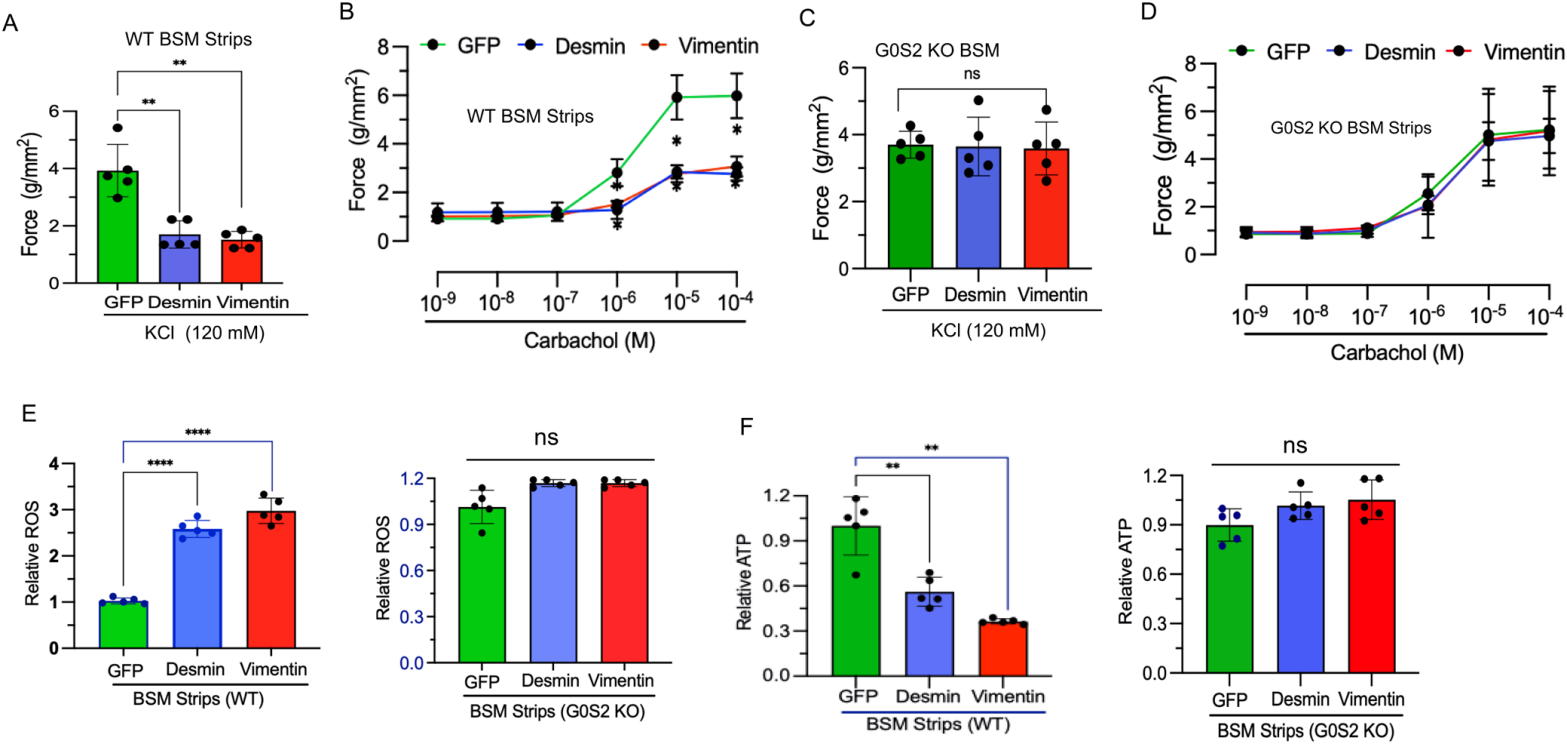
Increased expression of desmin and vimentin induces contractile and mitochondrial dysfunction via G0S2 in murine BSM tissue. BSM strips from WT and G0S2 KO mice were transduced with an adenovirus encoding GFP, desmin, and vimentin for 36 h. *BSM contraction*: (A–D) The contractile responses of murine BSM strips overexpressing desmin and vimentin in response to KCl (120 mM) and carbachol (10^−9^ to 10^−4^ M) stimulation were determined. (E) *Quantifications of ROS levels*: ROS levels in murine BSM strips were measured using isolated mitochondria with the Amplex Red Hydrogen Peroxide/Peroxidase Assay Kit. (F) *Quantifications of total cellular ATP levels*. ATP levels were measured based on firefly luciferin‐luciferase luminance intensity. Data are presented as mean ± SD and *n* = 5 in A–F.

To investigate the role of G0S2 in desmin‐ or vimentin‐overexpression‐induced mitochondrial dysfunction, we measured mitochondrial ROS and ATP levels in BSM strips from WT and G0S2 KO mice transduced with adenovirus encoding desmin or vimentin. G0S2 gene silencing restored mitochondrial ATP levels and reduced the elevated mitochondrial ROS caused by desmin and vimentin overexpression (Figure 11E,F).

### 3.9 Overexpression of Desmin and Vimentin Reduces Phospho MLC20 and Increases Phospho JNK Level via G0S2 in Human BSM Cells and Murine BSM Tissue

We next investigated the effect of desmin and vimentin overexpression on MLC20 phosphorylation in human BSM cells. The KCl‐induced MLC20 phosphorylation was attenuated in BSM cells overexpressing desmin and vimentin, relative to levels in GFP‐expressing human BSM cells (Figure 12A–C). G0S2 gene silencing prevented the reduction in pMLC20 levels induced by desmin and vimentin overexpression in human BSM cells (Figure 12A–C).

**FIGURE 12:**
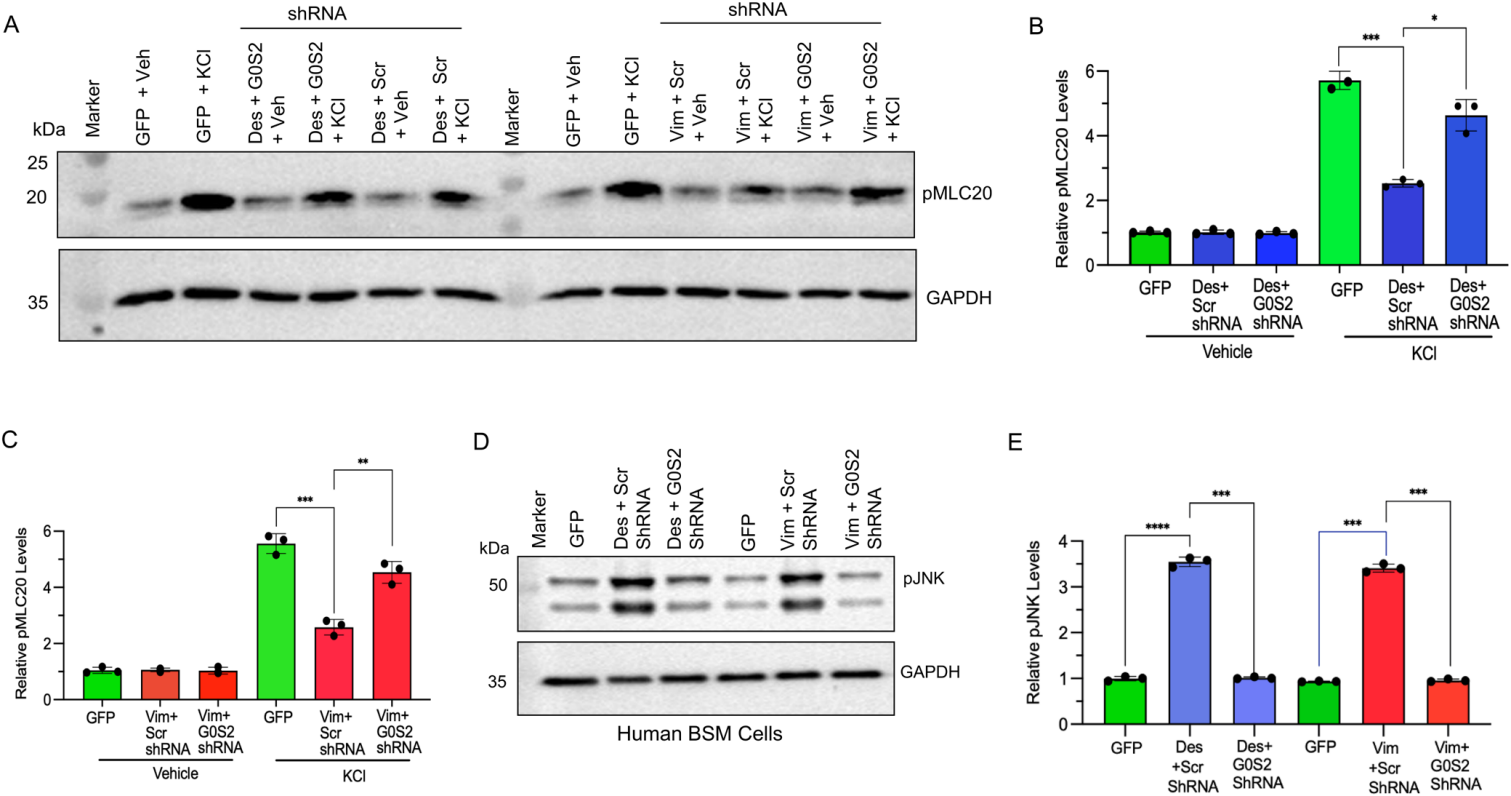
Overexpression of desmin and vimentin reduces phospho MLC20 and enhances phospho JNK levels via G0S2 in human BSM cells. Human BSM cells were transduced with adenovirus encoding GFP, desmin, and vimentin in the presence of scrambled (Scr) ShRNA or G0S2 ShRNA. (A) *Measurement of MLC20 phosphorylation*: BSM cells were stimulated with 120 mM KCl in the Tyrode buffer. Following stimulation, cells were lysed, and total protein extracts were subjected to immunoblotting for pMLC20 and GAPDH. GAPDH serves as the loading control. Representative immunoblots and their corresponding quantifications are shown in panels A–C. (D, E) *Measurement of phospho JNK levels*. Total protein lysates isolated from human BSM cells overexpressing desmin and vimentin were subjected to immunoblot analysis for pJNK and GAPDH. GFP‐expressing human BSM cells served as a control group. GAPDH was used as a loading control. Representative immunoblots and their corresponding quantifications are shown in panels D, E: In figure A–C, Veh (vehicle) refers to Tyrode buffer containing 2.7 mM KCl. Data are presented as mean ± SD and *n* = 3 in each group. *p < 0.05, **p < 0.01, ***p < 0.001 and ****p < 0.0001.

A previous study from our group demonstrated that the increased expression of desmin and vimentin induces pJNK levels in murine BSM tissue [16]. Therefore, we determined the relationship between G0S2 and pJNK in BSM cells overexpressing desmin or vimentin. The overexpression of desmin or vimentin in human BSM cells increased JNK phosphorylation relative to the levels in GFP‐expressing human BSM cells (Figure 12D,E), which was not observed in G0S2 knockdown cells (Figure 12D,E).

We next determined the effect of desmin and vimentin overexpression on MLC20 phosphorylation in murine BSM tissue. KCl‐induced MLC20 phosphorylation was attenuated in BSM strips overexpressing desmin and vimentin compared with GFP‐expressing BSM strips from WT mice (Figure 13A) and not in strips obtained from G0S2 KO mice (Figure 13B,C). Immunoblots analysis showed that overexpression of desmin and vimentin in murine WT BSM strips increased JNK phosphorylation relative to GFP‐expressing WT BSM strips (Figure 13D–F), but not in strips from G0S2 KO mice (Figure 13D–F). Collectively, our results demonstrate that desmin‐ and vimentin‐mediated modulation of pMLC20 and pJNK involves G0S2 in human BSM cells and murine BSM tissue.

**FIGURE 13:**
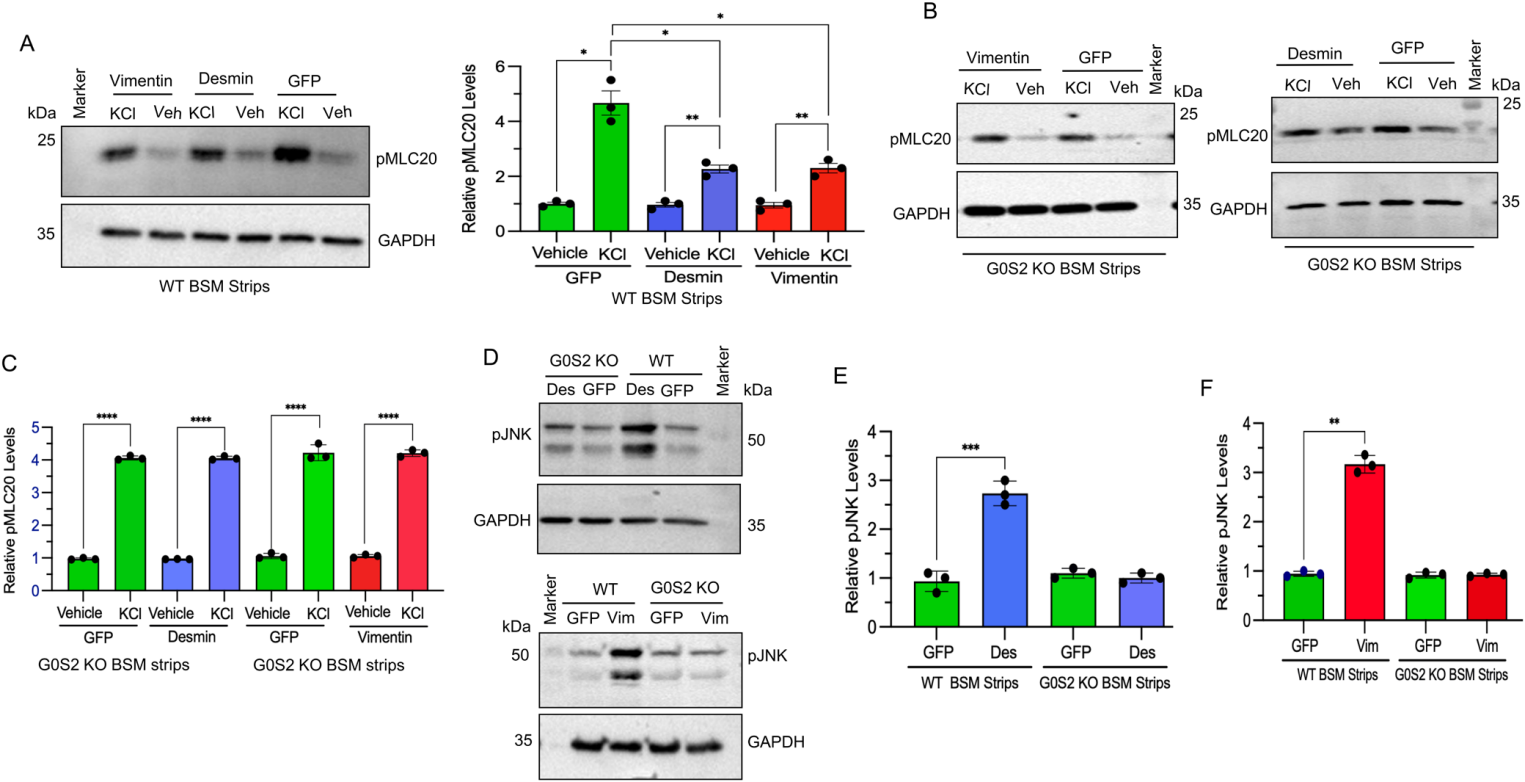
Overexpression of desmin and vimentin reduces phospho MLC20 and enhances phospho JNK levels via G0S2 in murine BSM tissue. BSM strips from WT and G0S2 KO mice were transduced with an adenovirus encoding GFP, desmin, and vimentin for 36 h. (A–C) *Measurement of phospho MLC20 levels*: Murine BSM strips overexpressing GFP, desmin, and vimentin were stimulated with 120 mM KCl in Tyrode buffer. Following stimulation, strips were frozen. Total protein isolated from these strips was subjected to immunoblotting for pMLC20 and GAPDH. GAPDH served as the loading control. Representative immunoblots and quantification of band intensities are shown in A–C. (D–F) *Measurement of phospho‐JNK levels*. Total proteins extracted from murine BSM strips overexpressing desmin and vimentin were subjected to immunoblotting for pJNK and GAPDH. GAPDH was used as a loading control. Representative immunoblots and their corresponding quantifications are shown in panels D–F. In figure A–C, Veh (vehicle) refers to Tyrode buffer containing 2.7 mM KCl. Data are presented as mean ± SD, with *n* = 3 mice in each group. *p < 0.05, **p < 0.01, ***p < 0.001 and ****p < 0.0001.

## 4 Discussion

A previous study from our group showed that desmin and vimentin overexpression reduced BSM contraction [16]. Our findings in the present study demonstrate that increased expression of desmin and vimentin in BSM results not only in an impact on contractile function but also causes mitochondrial impairment. BSM with elevated expression of desmin and vimentin generates less force and exhibits reduced levels of phosphorylated MLC20 compared to control BSM. Notably, increased expression of desmin and vimentin in human BSM cells reduces mitochondrial elongation, decreases ATP levels, and increases mitochondrial ROS levels. Microarray analysis revealed a significant induction of G0S2 mRNA expression, and G0S2 protein interacts with the mitochondrial protein VDAC in desmin‐ and vimentin‐overexpressing human BSM cells. Silencing G0S2 expression in BSM restored contractile function, pMLC20 levels, and ATP levels, and reduced the elevated mitochondrial ROS induced by desmin and vimentin overexpression. A previous study from our group demonstrated that desmin‐ or vimentin‐mediated attenuation of BSM contraction was associated with increased pJNK levels [16]. In this study, we demonstrate that desmin or vimentin overexpression increases pJNK and G0S2 levels and reduces BSM contraction. G0S2 gene silencing restored BSM contraction and phospho‐MLC20 levels, whereas reducing the phospho‐JNK levels in BSM overexpressing desmin and vimentin. These findings indicate that G0S2 regulates pJNK levels and suggest that G0S2 functions upstream of pJNK in mediating desmin‐ and vimentin‐induced BSM contractile dysfunction. Overall, these studies show that mitochondrial G0S2 mediates desmin‐ and vimentin‐induced contractile dysfunction and mitochondrial impairment in BSM.

Our studies also identified upregulation of the inflammatory cytokines IL‐6, IL‐8, and IL‐1β, and downregulation of CAV‐1 in BSM cells overexpressing desmin or vimentin. In support of these findings, prior studies have shown upregulation of the inflammatory cytokines IL‐6 and IL‐1β [48, 49] in animal models following PBOO. Previous studies have also shown downregulation of caveolins (CAV1, CAV2, and CAV3) in animal PBOO models and in human BPH‐induced BOO [14, 45, 50]. Studies using animal models of partial BOO have demonstrated increased expression of desmin and vimentin [13, 14, 51]. Stretch is the hallmark of PBOO. Studies using human BSM cells have demonstrated increased IL‐8 levels and enhanced JNK phosphorylation in response to stretch [52, 53]. Kushida et al. have shown that JNK kinase activity is upregulated and that pJNK levels increase in response to stretch in BSM [53]. Consistent with earlier observations, we show here that elevated levels of desmin and vimentin induce IL‐8 gene expression and increase phospho‐JNK levels in human BSM cells overexpressing desmin or vimentin. An unpublished study by our group shows that mechanical stretch induced overexpression of desmin and vimentin in human BSM cells. The gene and protein expression profiles, and protein kinase activation induced by desmin and vimentin overexpression closely recapitulate those observed in BOO, indicating that desmin and vimentin overexpression drive BOO‐induced gene expression and protein kinase activation rather than arising as a consequence of BOO.

Interestingly, overexpression of desmin or vimentin significantly altered mitochondrial dynamics and function. Overexpression of desmin and vimentin increases mitochondrial ROS, suggesting oxidative stress in BSM cells. Consistent with this observation, a study of obstructed bladders has shown that bladder dysfunction is associated with increased mitochondrial ROS levels [21]. Furthermore, to examine the causal relationship between desmin and vimentin proteins and mitochondrial oxidative stress, we evaluated their expression levels after treating cells with antimycin. Antimycin inhibits complex III activity in the mitochondrial electron transport chain, leading to ROS production. We examined the dose‐dependent effects of antimycin on desmin and vimentin expression in human BSM cells, which we used as an in vitro model of oxidative stress. Antimycin treatment did not significantly increase the expression of desmin and vimentin. Conversely, overexpression of desmin and vimentin induces mitochondrial ROS levels. Findings from previous and current studies suggest that desmin and vimentin overexpression drive oxidative stress, but oxidative stress does not induce their expression.

To characterize mitochondrial morphology, shape descriptors are commonly used in microscopic image analysis. The shape of mitochondria varies from spherical to a tubular network. Shape factors are computed from measured features such as length, area, and perimeter. The aspect ratio quantifies mitochondrial elongation and is computed as the ratio of the major to the minor axis. The form factor reflects both the elongation and branching complexity of mitochondria and is computed as *P*2/(4*πA*), where *P* is the perimeter and *A* is the area. The mitochondrial shape reflects the balance between mitochondrial fission and fusion.

The mitochondrial network refers to the interconnected distribution of mitochondria throughout a cell, rather than the shape of individual mitochondria. The network can be quantified using branching‐related parameters, such as branch count, branch length, and mean branch length. Mitochondrial morphology is tightly linked to function, with elongated and branched networks supporting efficient energy production [54, 55, 56, 57]. Prior studies of obstructed bladders have shown that bladder dysfunction is associated with impairment of mitochondrial structure and function [23, 24]. In this study, reduced elongation and branching observed in human BSM cells overexpressing desmin and vimentin can lead to reduced ATP levels and increased ROS, which may contribute to mitochondrial dysfunction.

Mitochondrial shape, movement, and distribution within cells are regulated by the highly coordinated interplay among mitochondrial fission, fusion, and dynamics. Mitofusin 1 (MFN1) and Mitofusin 2 (MFN2) are essential GTPases located on the outer mitochondrial membrane that promote mitochondrial fusion [58]. Decreased expression or deficiency of these proteins directly results in a severe reduction in mitochondrial fusion, leading to fragmented mitochondria characterized by decreased mitochondrial length or elongation [59]. Fis1 is a mitochondrial outer membrane protein that participates in the recruitment and regulation of fission machinery. It is one of the adaptor proteins that recruits the dynamin‐related protein 1 (DRP1), a cytosolic GTPase, to the mitochondrial outer membrane [60]. Increased Fis1 expression and DRP1 activation, specifically phosphorylation at Ser616, promote mitochondrial fission [60]. In this study, increased Fis1 expression and enhanced DRP1 phosphorylation at Ser616 in desmin‐ and vimentin‐overexpressing human BSM cells may promote fission and reduce elongation.

Emerging evidence supports the notion that the regulation of smooth muscle contraction extends beyond the conventional actomyosin interaction. It involves processes occurring within the cytoskeleton and at the interfaces between the cytoskeleton, the contractile apparatus, and the extracellular matrix [61]. New findings indicate that the dynamic reorganization of the subcellular filament network in both the cytoskeleton and the contractile apparatus is pivotal in the development and maintenance of smooth muscle force [61]. In this study, there were no obvious changes in actomyosin ultrastructure between control and desmin overexpression or between control and vimentin overexpression. However, due to dense packing of the filaments, minor changes in filament organization may not be detectable by this resolution. The observed reduction in MLC20 phosphorylation upon desmin and vimentin overexpression suggests impaired activation of the contractile pathway, thereby contributing to diminished contractility.

Our study has identified the mitochondrial protein G0S2 as an inducer of mitochondrial and contractile dysfunction in human BSM cells and murine BSM tissue that overexpresses desmin or vimentin. G0S2 was initially found to be differentially expressed in lymphocytes during the lectin‐induced transition from G0 to the G1 phase of the cell cycle [62, 63]. G0S2 is a multifunctional protein widely expressed and specifically abundant in liver, kidney, cardiac and skeletal muscle, brain, and adipose tissue [64]. This protein is found only in vertebrates, with no reported orthologs in other organisms [64]. The G0S2 gene is located on chromosome 1q32.2I in the human genome. The protein exhibits a highly conserved sequence showing significant similarity in the G0S2 amino acid sequence across different species. The gene contains cpG‐rich islands, and its upstream region includes tandem dinucleotide repeats [65]. G0S2 is a small protein consisting of 103 amino acids and is composed of an α‐alpha helical domain and a hydrophobic region, which may undergo conformational changes and adopt a β‐sheet structure [65].

G0S2 is localized to mitochondria [66] and plays a regulatory role in oxidative phosphorylation [67]. Previously, G0S2 was shown to interact with ATP synthase, specifically complex V (F0F1‐ATP synthase) of the OXPHOS system, and to stimulate ATP production in cardiomyocytes under hypoxic conditions [67]. In contrast, G0S2 has been shown to negatively regulate OXPHOS function in T cells [68], underscoring its context‐dependent role. In addition, a previous study has shown that G0S2 activates the AMPK signaling in vascular endothelial cells [69]. In human BSM cells, our findings indicate that G0S2 negatively regulates mitochondrial function in response to desmin and vimentin overexpression through direct interactions with VDAC. VDAC is considered a key metabolic gatekeeper of mitochondrial function. VDAC promotes ATP/ADP exchange as well as the flux of other metabolites across the mitochondrial outer membrane in association with [70, 71, 72, 73, 74] adenine nucleotide translocase [71, 75, 76] and mitochondrial creatine kinase [74, 77, 78]. Presumably, binding of G0S2 to VDAC obstructs ATP and ADP flux across the channel, thereby causing electron transport complexes to become metabolically starved of ADP, ultimately reducing ATP levels. Additionally, G0S2‐mediated JNK2 activation (increased phospho‐JNK levels) may reduce the BSM connection following desmin and vimentin overexpression. A previous study from our group demonstrated that desmin‐ or vimentin‐mediated attenuation of BSM contraction is associated with increased pJNK levels in murine BSM tissue [16].

In summary, our study demonstrates that increased expression of desmin and vimentin in BSM results in reduced ATP levels and increased ROS accumulation. BSM with elevated expression of desmin and vimentin generates less force and exhibits reduced levels of phosphorylated MLC20 compared to control BSM. Microarray analysis revealed a significant induction of G0S2 mRNA expression, and coimmunoprecipitation in desmin‐ and vimentin‐overexpressing human BSM cells showed a specific interaction between G0S2 protein and the mitochondrial channel protein VDAC. Silencing G0S2 expression in BSM restores contractility, phospho‐MLC20 levels, and mitochondrial ATP levels, whereas reducing the elevated mitochondrial ROS and phospho‐JNK levels induced by desmin and vimentin overexpression. Overall, these studies show, for the first time, that mitochondrial G0S2 mediates desmin‐ and vimentin‐induced contractile dysfunction and mitochondrial impairment in BSM.

## Author Contributions

E.B. conceived the study. E.B., E.J., C.T., K.T., A.B., S.‐S.S., S.A.S., E.L.S., S.S.A., R.B.P., and D.A.D. designed the study. E.B., E.J., C.T., K.T., J.H., S.A., D.M., F.S., J.M., V.D., P.D., and D.R.V. performed research and analyzed the data. I.G., Y.W., and J.G.T. provided G0S2 KO mice and reviewed the manuscript. E.B., E.J., C.T., and K.T. drafted the manuscript. E.L.S., S.S.A., R.B.P., and D.A.D. contributed to data analysis, reviewed the manuscript, and edited the manuscript. E.B. supervised the study and secured the funding. All authors reviewed and approved the final manuscript.

## Funding

This work was supported by HHS | NIH | NIDDK | Division of Diabetes, Endocrinology, and Metabolic Diseases (DEM) (R01DK129462).

## Disclosure

Generative artificial intelligence (ChatGPT Model 4.5, OpenAI; accessed in May 2025 and May 2026) served as a programming assistant while developing custom Python scripts for image analysis of mitochondrial morphology, ROS quantification, and filaments. The scripts employed well‐established image‐processing techniques. The authors did not provide study data or images to ChatGPT, and the authors determined the methods and analytical parameters used in the study. Thus, ChatGPT did not generate or select the underlying concepts and methods implemented in the scripts. The authors designed the computational workflow, identified the biological features and metrics to evaluate, and selected the methods and analytical parameters for the study. The authors used ChatGPT iteratively to translate established image‐processing techniques into Python code. ChatGPT also assisted with code implementation, computational optimization, and debugging. The authors reviewed, modified as needed, and validated all ChatGPT‐assisted code before use. ChatGPT was not used to develop the methodology, select approaches, interpret experimental results, or draw scientific conclusions. The authors retain full responsibility for the methodology, analysis, results, discussion, and overall conclusions presented in this manuscript.

## Conflicts of Interest

The authors declare no conflicts of interest.

## Supporting information


**Figure S1:** The list of differentially expressed transcripts exhibiting a 2‐fold or greater change between control and desmin‐overexpressing human BSM cells is shown. Differential expression analyzes were performed separately for desmin‐overexpressing and vimentin‐overexpressing BSM cells using a common control dataset; therefore, the same control data were used for comparisons of desmin‐ overexpression versus control and vimentin‐overexpression versus control.
**Figure S2:** The list of differentially expressed transcripts exhibiting a 1.8‐fold or greater change between control and vimentin‐overexpressing human BSM cells is shown. Differential expression analyzes were performed separately for desmin‐overexpressing and vimentin‐overexpressing BSM cells using a common control dataset; therefore, the same control data were used for comparisons of desmin‐ overexpression versus control and vimentin‐overexpression versus control.
**Table S1:** Primary and secondary antibodies used for immunoblotting, immunofluorescence and coimmunoprecipitation.

## Data Availability

All study data are included in the article.
