# Supplementary material for: Increased Expression of Desmin and Vimentin Impairs Mitochondrial Function and Reduces Contractility via G0S2 in Bladder Smooth Muscle
Source: FASEB J. 2026 Oct 21;40(20):e72380. doi: 10.1096/fj.202502912R (PMC13686112; doi:10.1096/fj.202502912R)
Supplement: Supplementary file 1 [file FSB2-40-e72380-s001.zip › 202502912R-sup-0006-SI_Text-S04.pdf]

Supplementary Fig S2

Figure Legend

The list of differentially expressed transcripts exhibiting a 1.8-fold or greater change between control and vimentin-overexpressing human BSM cells is shown. Differential expression analyses were performed separately for desmin-overexpressing and vimentin-overexpressing BSM cells using a common control dataset; therefore, the same control data were used for comparisons of desmin- overexpression versus control and vimentin-overexpression versus control.
