# Supplementary material for: Increased Expression of Desmin and Vimentin Impairs Mitochondrial Function and Reduces Contractility via G0S2 in Bladder Smooth Muscle
Source: FASEB J. 2026 Oct 21;40(20):e72380. doi: 10.1096/fj.202502912R (PMC13686112; doi:10.1096/fj.202502912R)
Supplement: Supplementary file 1 [file FSB2-40-e72380-s001.zip › Supplemental Table S1.doc]

| Serial Number | **Antibody** | **Cat Number** | **Vendor** |
| --- | --- | --- | --- |
| 1 | Desmin | ab 32362 | Abcam |
| 3 | SM22 | Ab10135 | Abcam |
| 4 | Desmin | D1033 | Sigma-Aldrich |
| 5 | MFN1 | ab57602 | Abcam |
| 6 | MFN2 | 9482T | Cell Signaling Technology |
| 7 | Smooth muscle Actin | Ab5694 | Abcam |
| 8 | COX IV | Ab16056 | Abcam |
| 9 | Vimentin | 5741S | Cell Signaling Technology |
| 10 | pDrp1 | 3455S | Cell Signaling Technology |
| 11 | OPA1 | 80471T | Cell Signaling Technology |
| 12 | pMLC20 | 3674S | Cell Signaling Technology |
| 13 | pJNK | 4668P | Cell Signaling Technology |
| 14 | VDAC | ab15895 | Cell Signaling Technology |
| 15 | Drp1 | 61113 | Abcam |
| 16 | Fis1 | **ALX-210-1037-0100** | Enzo Lifesciences |
| 17 | MYH11 | Ab125884 | Abcam |
| 18 | PINK1 | 6946T | Cell Signaling Technology |
| 19 | GAPDH | MAB374 | EMD Millipore |
| 20 | G0S2 | ZRB1813 | Sigma |
| 21 | G0S2 | Ab236113 | Abcam |
| 22 | Alexa Fluor 555 Anti-desmin | AB203422 | Abcam |
| 23 | Vimentin Cy3 | C9080 | Sigma |
| 24 | Rabbit HRP | Cat #111-035-003 | Jackson Immunological Research |
| 25 | Mouse HRP | Cat #115-035-003 | Jackson Immunological Research |
| 26 | Goat HRP | AB_10015289 | Jackson Immunological Research |

**Supplemental Table 1:** Primary and secondary antibodies used for immunoblotting, immunofluorescence and coimmunoprecipitation.
